# Spatiotemporal evolution of the clear cell renal cell carcinoma microenvironment links intra-tumoral heterogeneity to immune escape

**DOI:** 10.1186/s13073-022-01146-3

**Published:** 2022-12-19

**Authors:** Mahdi Golkaram, Fengshen Kuo, Sounak Gupta, Maria I. Carlo, Michael L. Salmans, Raakhee Vijayaraghavan, Cerise Tang, Vlad Makarov, Phillip Rappold, Kyle A. Blum, Chen Zhao, Rami Mehio, Shile Zhang, Jim Godsey, Traci Pawlowski, Renzo G. DiNatale, Luc G. T. Morris, Jeremy Durack, Paul Russo, Ritesh R. Kotecha, Jonathan Coleman, Ying-Bei Chen, Victor E. Reuter, Robert J. Motzer, Martin H. Voss, Li Liu, Ed Reznik, Timothy A. Chan, A. Ari Hakimi

**Affiliations:** 1 Illumina, Inc., 5200 Illumina Way, San Diego, CA 92122 USA; 2 Immunogenomics and Precision Oncology Platform, Memorial Sloan Kettering Cancer Center, New York, NY 10065 USA; 3 Department of Pathology, Memorial Sloan Kettering Cancer Center, New York, NY 10065 USA; 4 Department of Medicine, Genitourinary Oncology, Memorial Sloan Kettering Cancer Center, New York, New York, NY 10065 USA; 5 Human Oncology and Pathogenesis Program, Memorial Sloan Kettering Cancer Center, New York, NY 10065 USA; 6 Urology Service, Department of Surgery, Memorial Sloan Kettering Cancer Center, New York, NY 10065 USA; 7 Department of Surgery, Head & Neck Service, Memorial Sloan Kettering Cancer Center, New York, NY 10065 USA; 8 Interventional Radiology, Memorial Sloan Kettering Cancer Center, New York, NY 10065 USA; 9 Computational Oncology Service, Memorial Sloan Kettering Cancer Center, New York, NY 10065 USA; 10 Marie-Josée and Henry R. Kravis Center for Molecular Oncology, Memorial Sloan Kettering Cancer Center, New York, NY 10065 USA; 11 Department of Radiation Oncology, Memorial Sloan Kettering Cancer Center, New York, NY 10065 USA; 12 Center for Immunotherapy and Precision Immuno-Oncology, Cleveland Clinic, Cleveland, OH 44195 USA; 13 Lerner Research Institute, Cleveland Clinic, Cleveland, OH 44195 USA; 14 National Center for Regenerative Medicine, Cleveland Clinic, Cleveland, OH 44195 USA

## Abstract

**Background:**

Intratumoral heterogeneity (ITH) is a hallmark of clear cell renal cell carcinoma (ccRCC) that reflects the trajectory of evolution and influences clinical prognosis. Here, we seek to elucidate how ITH and tumor evolution during immune checkpoint inhibitor (ICI) treatment can lead to therapy resistance.

**Methods:**

Here, we completed a single-arm pilot study to examine the safety and feasibility of neoadjuvant nivolumab in patients with localized RCC. Primary endpoints were safety and feasibility of neoadjuvant nivolumab. Then, we spatiotemporally profiled the genomic and immunophenotypic characteristics of 29 ccRCC patients, including pre- and post-therapy samples from 17 ICI-treated patients. Deep multi-regional whole-exome and transcriptome sequencing were performed on 29 patients at different time points before and after ICI therapy. T cell repertoire was also monitored from tissue and peripheral blood collected from a subset of patients to study T cell clonal expansion during ICI therapy.

**Results:**

Angiogenesis, lymphocytic infiltration, and myeloid infiltration varied significantly across regions of the same patient, potentially confounding their utility as biomarkers of ICI response. Elevated ITH associated with a constellation of both genomic features (HLA LOH, CDKN2A/B loss) and microenvironmental features, including elevated myeloid expression, reduced peripheral T cell receptor (TCR) diversity, and putative neoantigen depletion. Hypothesizing that ITH may itself play a role in shaping ICI response, we derived a transcriptomic signature associated with neoantigen depletion that strongly associated with response to ICI and targeted therapy treatment in several independent clinical trial cohorts.

**Conclusions:**

These results argue that genetic and immune heterogeneity jointly co-evolve and influence response to ICI in ccRCC. Our findings have implications for future biomarker development for ICI response across ccRCC and other solid tumors and highlight important features of tumor evolution under ICI treatment.

**Trial registration:**

The study was registered on ClinicalTrial.gov (NCT02595918) on November 4, 2015.

**Supplementary Information:**

The online version contains supplementary material available at 10.1186/s13073-022-01146-3.

## Background

Clear cell renal cell carcinoma (ccRCC) is the most common histological subtype of kidney cancer and demonstrates a high response rate to immune checkpoint inhibitors such as nivolumab, pembrolizumab, and ipilimumab [1–4]. However, only a subset of ccRCC patients respond to ICI, and biomarkers for ICI response in other disease settings such as tumor mutation burden, neoantigen load, and mismatch repair deficiency do not associate with ICI response in ccRCC [5–8]. Recently, several studies have identified transcriptomic microenvironmental features including angiogenic gene expression, T cell infiltration, and myeloid activation that correlate with response or resistance to ICI and combination therapies in ccRCC [8–14]. This suggests that the ccRCC microenvironment, in addition to genomic factors, influences ICI response.

In parallel, recent work has demonstrated the prevalence of ITH in untreated ccRCC [15]. This study has largely focused on heterogeneity in the presence of key driver mutations and copy number alterations and has demonstrated that ccRCC tumors follow one of a small number of evolutionary trajectories, each of which is associated with distinct patterns of genomic ITH and clinical prognosis. However, the potential for non-genomic heterogeneity in the tumor microenvironment, including but not limited to variability in the amount and identity of immune cells in spatially distinct regions of the same tumor is overlooked. Recently, we and others described substantial heterogeneity in the tumor microenvironment (TME) in several small cohorts of ccRCC tumors both in the treatment-naïve and treatment-exposed settings, raising the possibility that heterogeneity in the TME may itself shape the evolution of the tumor and its likelihood to respond to therapy [16, 17].

**Fig. 1:**
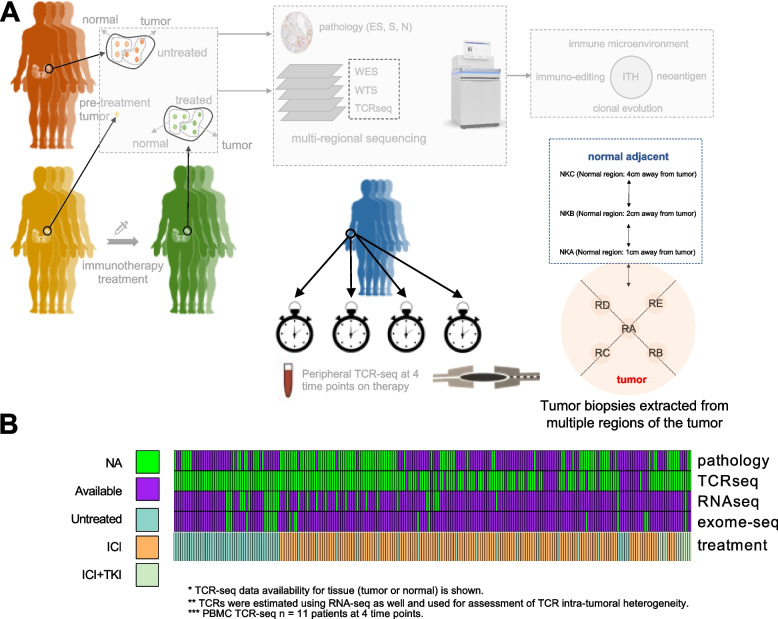
Patient characteristics and study design. **A** Multi-regional multi-omics was performed on 29 patients. Serial and multi-regional sampling strategies shown schematically. **B** In total, 6 out of 29 patients were untreated and the rest were treated with ICI or in combination with TKI. TCRseq of PBMC was performed at 4 time points on therapy for a subset of patients. In addition, pathological review was performed to assign N-TIL (tumors sparsely infiltrated by TILs), S-TIL (tumors dominated by stromal TILs), and ES-TIL (tumors with substantial levels of both epithelial and stromal TILs) classes to a subset of patients

In this study, we hypothesized ccRCC tumors with elevated ITH constitute a genomically and immunologically distinct class of tumors, with distinguishing clonal/subclonal genomic alterations, immunologic profiles, and therapeutic response trajectories. To test this hypothesis, we utilize whole-exome sequencing (WES), whole-transcriptome sequencing (WTS), TCRseq, and histopathologic multi-regional data across a cohort of untreated and ICI-exposed patients from a phase 2 clinical trial to reveal the molecular determinants of therapy response in ccRCC (Fig. 1 and Additional file 1: Table S1). Our integrated analysis demonstrated that ITH is highly correlated among genomic, transcriptomic, and TME characteristics. ITH-high tumors are enriched for features including SETD2 and PBRM1 mutations, HLA loss of heterozygosity (HLA LOH), and CDKN2A/B loss. Immunologically, ITH-high tumors display a depletion of putative neoantigens, elevated myeloid activation, and reduced T cell diversity that are in aggregate associated with escape from the anti-tumor immune response. Premised on these observations, we developed a transcriptional signature for immune escape which correlates with distinct histopathologic patterns and is associated with ICI resistance across several diverse clinical trial cohorts.

## Methods

### Sample acquisition

After acquiring informed consent and institutional review board approval from Memorial Sloan Kettering Cancer Center (MSK), partial or radical nephrectomies were performed at MSK (New York) and stored at the MSK Translational Kidney Research Program (TKRCP). Samples were flash frozen and stored at − 80 °C prior to molecular characterization. Clinical metadata was recorded for all tumor samples. All patients represent clear cell histology and were treated via ICI alone or in combination with tyrosine kinase inhibitor (TKI). All treatments were administered prior to surgery in a neoadjuvant setting and biopsies were collected. Detailed clinical data and treatment regimen for each patient is included in Additional file 1: Table S2. After obtaining informed consent for tissue collection, samples were directly obtained from the operating room during nephrectomy. At the time of specimen extraction, samples of around 1–1.5 cm were obtained by the treating surgeon (A. A. H.) from spatially distinct tumor regions (at least 1 cm apart) and each one labeled according to its spatial location (relative to the adjacent kidney). For untreated patients (MR…) Additional file 1: Table S2, 3–5 tumor regions and an adjacent normal kidney sample (at least 2 cm away from the tumor) were obtained. For all ICB-treated patients (Nivo…SC…), three to five tumor regions, an adjacent normal kidney sample (at least 2 cm away from the tumor), and PBMC sample (in cellular preparation tubes, CPTs) were obtained.

### Untreated cohort

Using and institutional database, we identified six patients with advanced or metastatic ccRCC that underwent nephrectomy with multi-regional data available, MR01,02,03,05,06, SC03. Clinical and pathologic data is available in Additional file 1: Table S2.

### Neoadjuvant multi-regional cohort

This open-label, single-arm, pilot prospective study was done at Memorial Sloan Kettering Cancer Center and funded through the National Institute of Health’s Cancer Therapy Evaluation Program (CTEP) [1]. Primary endpoints were safety and feasibility of neoadjuvant Nivolumab. Patients received nivolumab (dose initially 3 mg/kg, then protocol amended to 240 mg flat dose) every 2 weeks for 4 treatments. Surgery was planned 7–14 days after the last dose. Prior to starting therapy, all patients had a kidney biopsy to confirm ccRCC, and tumor staging with renal protocol MRI and CT of the chest. After 4 doses and prior to surgery, patients also had a renal protocol MRI. Changes in primary tumor size were assessed according to Response Evaluation Criteria in Solid Tumors (RECIST) version 1.1. Resection of the primary tumor and lymph nodes was done according to standard institutional procedures. From May 27, 2016, to September 9, 2019, 21 patients were screened and 18 were enrolled into the study of which 17 had available genomic data. Baseline patient characteristics are in Additional file 1: Table S2. All patients had localized disease at time of enrollment and biopsy-proven clear cell RCC. Perioperative and pathological details are included in Additional file 1: Table S2. Median time to nephrectomy after the last dose of nivolumab was 10.5 days (range, 9–13 days). In this small group of patients who had cancer confined to the kidney, this approach appeared safe and feasible. The details and clinical outcome of this trial are currently published in [1].

### Metastatic multi-regional cohort

Using an institutional database, we identified 6 additional patients who had received ICI prior to nephrectomy (Additional file 1: Table S2). All patients had metastatic disease at time of ICI; two received anti-VEGF therapies before ICI.

### Therapy response collection

All ICI-treated patients (NIVO) were only neoadjuvant treated and no responses were seen on imaging for any of the patients.

SC06: ipilimumab/nivolumab resistant (i.e., stable disease).

SC08: ipilimumab/nivolumab mixed response (partial response).

SC09: sutent then ipilimumab/nivolumab—progression of disease (progressed disease).

SC10: ipilimumab/nivolumab (complete response).

SC12: ipilimumab/nivolumab resistant (i.e., stable disease).

SC14: ipilimumab/nivolumab resistant then lenvatinib/pembrolizumab-resistant (i.e., stable disease).

### Multi-regional sampling

For the prospective neoadjuvant trial and the “MR” samples, single-region biopsies were obtained preoperatively. Following nephrectomy, tumors were bivalved and 5 regions were chosen: One region from the tumor center and 4 from each quadrant (upper medial, upper later, lower medial, lower lateral). Grossly necrotic or hemorrhagic regions were avoided. For the remaining samples (those treated with definitive immunotherapy “SC”) regions were taken from distinct regions of tumors separated by 1–2 cm avoiding grossly necrotic or hemorrhagic regions).

### Whole-exome sequencing

Libraries for whole-exome sequencing were generated with TruSight Oncology DNA Library Prep Kit (V1) with 40 ng input DNA per sample. TruSight Oncology index PCR products were directly used for enrichment, and target exome enrichment was performed using the IDT xGen Universal Blockers and IDT xGen Exome Research panel V1. A single-plex hybridization was done overnight at 65 °C. Accuclear dsDNA Ultra High Sensitivity assay (Biotium) was used for library quantification of the post-enriched libraries. Post enrichment libraries were normalized using bead-based normalization and pooled. Samples were sequenced with 101 bp paired-end reads on Illumina NovaSeq™ 6000 S4 flow cell using the XP workflow for individual lane loading (12-plex per lane). On average, each sample yielded 500 million reads and MEDIAN_TARGET_COVERAGE depth of 360X.

### Whole-transcriptome sequencing

Libraries for whole-transcriptome RNAseq were generated with Illumina TruSeq Stranded Total RNA. One hundred nanograms RNA was used as input for Ribo-Zero rRNA Removal Kit, with Illumina TruSeq RNA UD Indexes (96 indexes) for sample indexing. Qubit dsDNA High Sensitivity assay (Thermo Fisher Scientific) was used for library quantification. Sequencing was done on Illumina NovaSeq™ 6000 S2 (36-plex) or S4 (72-plex) flow cell with 76 bp paired-end sequencing to produce ~ 200 million paired reads per library.

### T cell repertoire sequencing

Libraries for T cell repertoire sequencing were generated with AmpliSeq for Illumina Library PLUS paired with AmpliSeq cDNA Synthesis for Illumina with 100 ng RNA input per cDNA synthesis reaction. The TCR beta-SR Panel was used for generating amplicons, and AmpliSeq CD Indexes Set A for Illumina were used for sample barcodes. Qubit dsDNA High Sensitivity assay (Thermo Fisher Scientific) was used for library quantification. Sequencing was done on the NextSeq 550 (41-plex) with 151 bp paired-end sequencing to produce ~ 5 million paired reads per library.

### WTS pipeline

WTS raw read sequences were aligned against human genome assembly hg19 by STAR 2-pass alignment [18]. QC metrics, for example general sequencing statistics, gene feature, and body coverage, were then calculated based on the alignment result through RSeQC. WTS gene-level count values were computed by using the R package GenomicAlignments [19] over aligned reads with UCSC KnownGene [20] in hg19 as the base gene model. The union counting mode was used and only mapped paired reads after alignment quality filtering were considered. Finally, gene-level FPKM (fragments per kilobase million) and raw read count values were computed by the R package DESeq2 [21].

### ESTIMATE

The ESTIMATEScore, which is the estimate of the presence of stromal and immune cells in tumor tissue, is calculated through the ESTIMATE R package [22] based on a given gene expression profile in FPKM.

### Immune deconvolution analysis

Two distinct popular computational methods, ssGSEA [23] and CIBERSORT [24], were chosen for immune deconvolution analysis. Signature gene lists of immune cell types for ssGSEA were obtained from Bindea et al. [25] and Senbabaoglu et al. [4]. ssGSEA takes the sample FPKM WTS expression values as the input and computes an enrichment score for the given gene list of immune cell type relative to all other genes in the transcriptome. On the other hand, CIBERSORT also takes FPKM WTS expression values as the input but uses a signature gene expression matrix of interest immune cell types instead to compute the infiltration level of each immune cell type. The LM22 immune cell signature which was validated and published along with CIBERSORT is used. We also used FRICTION [26] to deconvolute WTS into absolute CD8 and CD4 T cells as well as CD19 B cells.

### HERV quantification

We used WTS to quantify HERVs as described before [26]. Briefly, all WTS reads were aligned (using STAR aligner with optimized multi-mapping options) to a custom genome build where human reference (hg19) and HERV-specific reference are combined. Then reads aligned to non-HERV genes are removed and the rest are annotated. Three samples contained super high median HERVs (Grubbs test *P* < 0.05) and removed for better visualization.

### WES analysis pipeline

Raw sequencing data were aligned to the hg19 genome build using the Burrows-Wheeler Aligner (BWA) version 0.7.17 [27]. Further, indel realignment, base-quality score recalibration, and duplicate-read removal were performed using the Genome Analysis Toolkit (GATK) version 3.8 [28] following raw read alignment guidelines [29]. VarScan 2 [30], Strelka v2.9.10 [31], Platypus 0.8.1 [32], Mutect2—part of GATK 4.1.4.1 [29], Somatic Sniper version 1.0.5.0 (SNVs only), and [33] were used for small variant calling, and combination of 2 out 5 callers is reported as per Cancer Genome Atlas Research Network recommendations [34]. Variants were filtered using the following criteria:

1. Tcov (tumor coverage) > 10 and Taf (tumor allele frequency) ≥ 0.04 and Ncov (normal coverage) > 7 and Naf (normal allele frequency) ≤ 0.01 and Tac > 4 are set to PASS
2. Common SNPs are eliminated by comparison to snp142.vcf
3. Rare variants found in dbSNP are kept if Naf = 0
4. Variants with Tcov < 20 or Tac < 4 are marked as low_confidence
5. Only variants called by more than 1 caller are reported.
6. Common variables gnomAD v 2.1.1 are excluded.

Variants were annotated using Ensembl Variant Effect Predictor (VEP) [35]. Additional optimization and filtering are applied for INDELS. INDELS in blacklisted regions (https://www.encodeproject.org/annotations/ENCSR636HFF/) and low mappability regions (such as repeat maskers) are excluded as per [36]. Combination of filtered SNV and INDELS are used by maftools R package to generate oncoplots and summary plots, as per author’s recommendations (https://www.bioconductor.org/packages/release/bioc/vignettes/maftools/inst/doc/maftools.html).

All nonsynonymous point mutations identified as above were translated into strings of 17 amino acids with the mutant amino acid situated centrally using a bioinformatics tool called NAseek. A sliding window method is used to identify the 8–11 amino acid substrings within the mutant 17-mer that had a predicted MHC Class I binding affinity of ≤ 2%Rank to one (or more) of the patient-specific HLA alleles. Binding affinity for the mutant and corresponding wild type nonamer is analyzed using NetMHCpan4.0 software. Only neoantigens with TPM > 1 are considered to be expressed.

Allele-specific copy number analysis is done by the FACETS v.6.1 [37]. Allele-specific HLA loss is determined using LOHHLA as described before [38].

### RNA and TCR ITH scores

Gene- and patient-wise intra-patient heterogeneity scores were calculated using multi-region data. Data was first median-centered to remove any gene-level bias. For each gene, the difference between each pair of samples from the same tumor was calculated. The median difference between the paired-differences was then taken, yielding a gene-specific, patient-specific measure of heterogeneity. This was repeated for all genes, across all tumors, generating a matrix of gene by patient values. Gene intratumor heterogeneity values are summarized as the median value per gene across all tumors in the cohort. Patient intratumor heterogeneity values are summarized as the median value per tumor across all genes. Patient intratumor heterogeneity values represent the expected value of the absolute log2-fold change for a randomly chosen gene within a given tumor.

TCR ITH score is defined as 1 − percentage of shared clonotypes across multiple regions of tumor based on WTS. T cell clones are estimated using MiXCR application on Illumina BaseSpace (http://basespace.illumina.com/apps/). Furthermore, all ITH scores are classified as high versus low using the median as threshold.

### Distinction between dedicated TCRseq and TCR clones inferred from RNAseq using MiXCR

All TCR-associated data analysis in this study (including tissue or PBMC) are based on ultra-deep T cell repertoire sequencing (targeted TCRseq) to mitigate undersampling of T cell clones except TCR ITH analysis in Fig. 3B where ITH associated with multi-regional sequencing is derived from MiXCR T cell estimates from RNAseq data due to the lack of multi-regional TCRseq for all patients.

### ccRCC evolutionary subtypes and intratumor DNA heterogeneity score

DNA ITH score is calculated as the ratio of subclonal to clonal driver genomic alterations including SNVs, INDELs, and SCNA [15]. A genomic alteration is defined to be subclonal if it is present in less than half of the regions collected in each patient. Patients with enough DNA biopsies collected are classified into 1 of the 7 ccRCC evolutionary subtypes as described before [15]. We used neighbor joining tree construction in ape package in R [39] for reconstruction of tumor clones. TCGA ITH score was obtained from a previous study as measured by the number of clones estimated per sample using PhyloWGS [40]. Briefly, PhyloWGS is a method to infer tumor evolution evolutionary using the relationships between tumor subpopulations based on variant allele frequencies while considering copy number alterations.

### HLA and TCR diversity

Shannon entropy is calculated to define TCR diversity [41]. We used MiXCR application on Illumina BaseSpace (http://basespace.illumina.com/apps/) for alignment and T cell clonotype identification. Immunarch (https://immunarch.com/) [42] was used for downstream analysis including visualization and data analysis. Morisita index [43] was used to measure clonotype overlap. HLA diversity index is measured as adopted from [44] as described in [26].

### Neoantigen depletion

The fraction of neoantigens depleted is defined for each sample where pre-treatment data was available. We first calculated the neoantigen depletion as the number of neoantigens that were undetectable after therapy but were detected pre-treatment. The fraction of neoantigens depleted was then defined as the ratio of the total number of depleted neoantigens over total pre-treatment neoantigens. To distinguish neoantigen depletion due to contraction (immune elimination) from evasion, we exclude any neoantigens that were depleted without the presence of HLA LOH (defects in antigen presentation machinery), or reduced expression, i.e., log2(FC) <  − 1 where FC is the fold change defined as the ratio of posttreatment TPM over pre-treatment TPM after correction for tumor purity. Conversely, a neoantigen is annotated and was deleted due to immune elimination if log2(FC) >  = 0, and no HLA LOH was detected. Likewise, HERV editing is defined as the median change in the expression of immunogenic HERVs compared to pre-treatment expression. Immunogenic HERVs refer to HERV loci whose expression strongly correlates with TIL abundance, FDR < 0.05.

### Weighted gene co-expression network analysis (WGCNA) and gene signature extraction

We performed WGCNA [44] on all samples where the fraction of neoantigens depleted was available similar to previously described [11]. Briefly, genes with low expression values and invariant genes, that is, genes that were expressed in < 5% of samples or had s.d. ≤ 1 for expression (log2 TPM), were filtered together with non-coding genes. The soft power of 6 was chosen based on goodness of fit to a scale-free network. We first annotate modules as JAVELIN or angiogenesis according to the Spearman correlation between the module eigengene and JAVELIN or angiogenesis ssGSEA scores (highest correlation is classified as JAVELIN or angiogenesis module). Likewise, among all modules, the module with the highest Spearman correlation with the fraction of neoantigens depleted was annotated as immune escape module (85 genes). This 85 genes’ gene signature was strongly associated with PFS of Avelumab plus Axitinib in JAVELIN Renal 101 (HR = 1.45, *P* = 0.02, Additional file 2: Fig. S1 A). To further refine this gene signature, we first sorted genes based on their pairwise Spearman correlation (Additional file 2: Fig. S1 B) and then selected genes with the highest Spearman correlation such that no genes have a Spearman correlation < 0.6 (Additional file 2: Fig. S1 C). This reduced the number of genes to total of 12 highly correlated genes known as immune escape signature (TIMP1, PXDN, COL15A1, OLFML2B, COL5A2, DLX5, SOX11, KLHDC8A, UNC5A, ADAMTS14, MMP11, FN1). Several genes (ADAMTS14, MMP11, FN1, COL5A1, COL5A2, and TIMP1) in this signature have previously been described as TGF-β-associated extracellular matrix genes that are linked to immune evasion and immunotherapy failure [45].

### TCGA validation of CDKN2A/B loss with ITH and myeloid enrichment

We downloaded WES data from TCGA KIRC cohort from https://www.cancer.gov/tcga and processed the raw data using the same pipeline used to process data generated for the multi-regional cohort as described earlier. CDKN2A/B loss calls extracted and the association with ITH assessed. TCGA ITH score was obtained from a previous study as measured by the number of clones estimated per sample using PhyloWGS [40]. Likewise, to evaluate the association between CDKN2A/B loss and myeloid score, gene expression count data corresponding to TCGA KIRC cohort was downloaded from https://www.cancer.gov/tcga and ssGSEA of myeloid signature calculated as described earlier.

### scRNAseq data analysis pipeline

scRNAseq data was downloaded from https://www.ncbi.nlm.nih.gov/sra/PRJNA705464. This repository contains a Seurat object containing the raw counts, normalized counts, dimensionality reduction post-batch correction, and cell type identities based on Krishna et al. study [16], which was used for further analysis in this study.

### Statistical analysis

All statistical tests were performed in R. To calculate correlations, cor.test with Spearman’s method was used. Tests comparing distributions were performed using wilcox.test. All statistical analyses were two-sided, and *p*-values were Benjamini–Hochberg corrected.

## Results

### The landscape of microenvironmental ITH in ccRCC

To study ITH in ccRCC, we completed ultra-deep (median coverage of 360X) multi-regional whole-exome sequencing and whole-transcriptome sequencing across 142 tumor regions from 29 patients, including 6 untreated and 23 post ICI (see “Methods” and Additional file 1: Table S2. The details and clinical outcome of this trial are currently published in [1]). Tumor biopsies were extracted from different regions of the same primary tumor unless specified (Fig. 1A, B, Additional file 1: Table S2). While intratumoral genetic heterogeneity in ccRCC is well-described [46], comparatively little is known about the extent of microenvironmental heterogeneity and its relationship to other molecular features of the tumor. To measure the extent of intratumoral microenvironmental heterogeneity, we leveraged multi-regional WTS of up to 5 regions from 29 patients. Using single-sample gene set enrichment analysis (ssGSEA) of established gene signatures, we quantified the expression of several TME gene expression signatures recently proposed as biomarkers of response to ICIs and antiangiogenic agents [47] (myeloid signature [9], JAVELIN signature [11], and angiogenesis signature, see “Methods” and Additional file 1: Table S3). We confirmed that these RNA signatures accurately quantified the abundance of key immune populations using matched immunofluorescence data, including statistically significant associations between CD31/angiogenesis (*p* = 0.0003), CD8/JAVELIN T cell signature (*p* = 0.02), and CD68/Myeloid infiltration (*p* = 0.0013) (Additional file 2: Fig. S2).

**Fig. 2:**
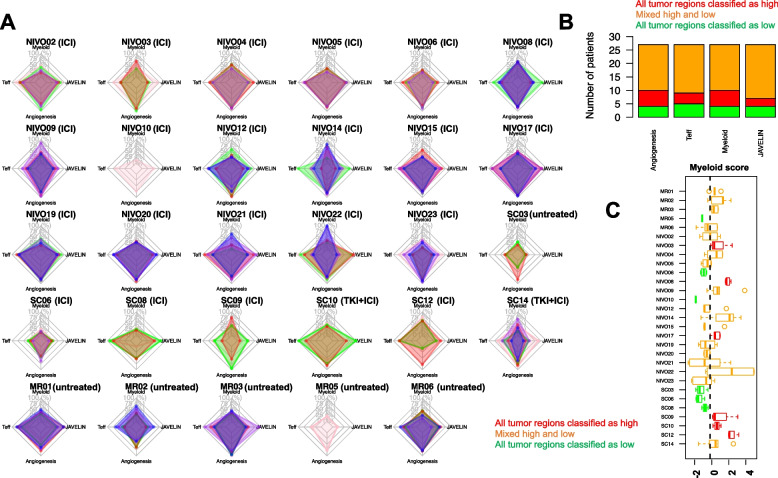
TME ITH in ccRCC. **A** Intratumoral heterogeneity of several gene expression signatures across multiple tumor regions. For each patient, each tumor region is assigned a unique color. For each vertex on the radar plot, we plot the *Z*-score of the relevant RNA signature relative to all other samples in the cohort. Min and max radius for each feature in each panel represent min and max of that feature across the cohort. A wide (narrow) spread of dots for a given feature in a single patient corresponds to qualitatively large (small) heterogeneity of that microenvironmental RNA signature in that patient. **B** For each gene signature, the number of patients who were classified as high or low or a mixture of high and low across tumor regions are shown. Two patients (MR05 and NIVO10) were excluded since WTS data of only one region was available. Also, pre-treatment regions of ICI-treated patients were excluded to avoid treatment-related effects in these signatures. **C** Intratumoral heterogeneity of myeloid score observed across multiple regions of tumors of patients in this study

For each RNA signature, we normalized scores to capture the magnitude of expression relative to all other profiled regions in our cohort. We then investigated the variability of microenvironmental RNA signatures across regions, finding that they demonstrated extensive heterogeneity across tumor regions from the same patient (Fig. 2A). While a small number of patients showed relatively uniform immune infiltration (e.g., NIVO02, Fig. 2A), the significantly more common phenomenon was for patients to exhibit regions both above and below the median score for a microenvironmental feature of interest (e.g., angiogenesis in MR03, JAVELIN/T-effector signatures in NIVO22).

ITH in expression signatures had substantial consequences on the accuracy of stratifying patients into high/low expressing groups based on a single-region biopsy. Using the myeloid signature (which has previously been associated with poor response to ICI [9]), we calculated the median myeloid score across all samples in the cohort and then assigned each tumor region to either a high myeloid or low myeloid group. Consequently, we observed that the majority of patients had tumor regions classified into both myeloid-high and myeloid-low regions (Fig. 2C). Given that several of these signatures are under active investigation as biomarkers of response to ICI, we investigated more generally how classification of regions into high/low was affected by ITH. Remarkably, in more than half of the patients, clinically relevant signatures (Angiogenesis, T-effector, Myeloid, and JAVELIN) could not be consistently classified as high or low (Fig. 2B, 2 patients, i.e., MR05 and NIVO10 were excluded since WTS data of only one region was available).

**Fig. 3:**
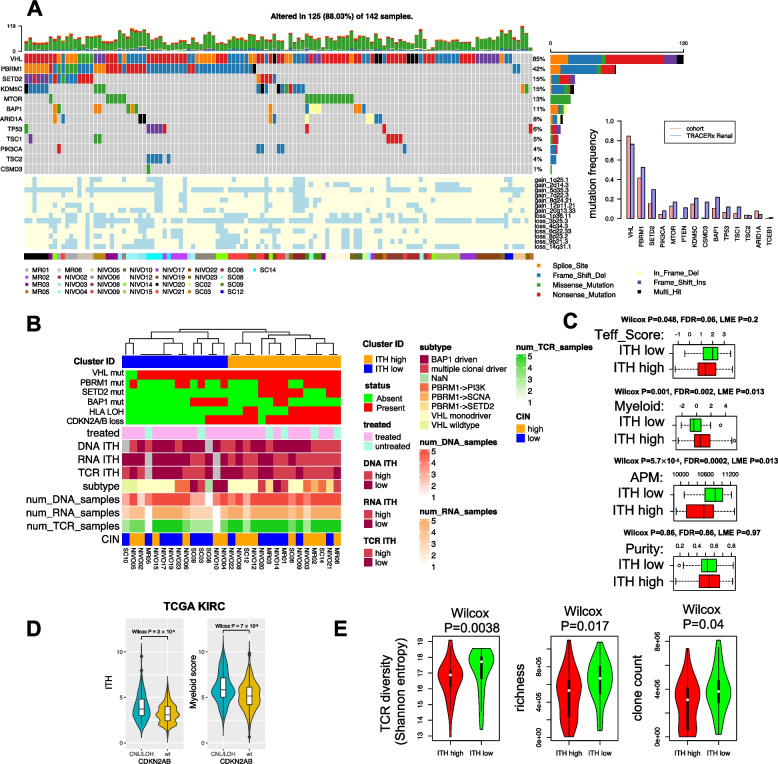
Landscape of ITH in ccRCC. **A** Oncoprint of key ccRCC driver mutations and copy number alterations for all regions of all 29 patients in this cohort. Margin shows comparison between mutation frequency observed in this cohort and TRACERx Renal. **B** We have performed unsupervised hierarchical clustering of genomic features including patient level presence or absence of a small variant in VHL, PBRM1, SETD2, BAP1 (most commonly mutated genes) as well as loss of heterozygosity in HLA genes as well as 9p (which includes CDKN2A/B) SCNA which are known to affect ICI response. Heatmap shows ITH high vs low classification across data type. Annotation illustrates evolutionary subtypes and treatment status of patients. A patient is annotated as wildtype if all regions are wild type for that alteration. Cases where ITH score could not be calculated due to lack of sufficient number of biopsies are shown in gray pixels. CIN: chromosome instability. **C** Association between antigen presentation machinery (APM), effector T cell (Teff) and myeloid gene signatures, and ITH. Wilcox *P*, false discovery rate (FDR), and linear mixed effect (LME) *P* shown. **D** Intratumoral heterogeneity and myeloid score are associated with CDKN2A/B loss in TCGA KIRC cohort. **E** ITH low patients show a significantly higher TCR diversity, richness, and clone count

We hypothesized elevated microenvironmental heterogeneity may reflect the presence of underlying genomic driver alterations. To test this, we leveraged multi-regional WES data collected for these patients. Frequencies of established ccRCC driver alterations were in agreement with a previous multi-regional study by TRACERx Renal [15] (Fig. 3A). We performed unsupervised hierarchical clustering of major ccRCC driver mutations (with mutation frequency > 10% across TCGA KIRC cohort and TRACERx Renal [15]) that were previously shown to be associated with ITH [15] (i.e., *VHL*, *PBRM1*, *SETD2*, *BAP1*) and genomic alterations enriched with metastatic disease and ICI response (HLA LOH and CDKN2A/B copy number loss) [38, 44, 48], ultimately identifying two clusters (Fig. 3B, Additional file 1: Table S4). We compared the results of these clusters to aggregate, univariate measures of intratumoral DNA, RNA, and T cell receptor (TCR) heterogeneity. Interestingly, one cluster was characterized both by an enrichment of specific genomic alterations (SETD2 mutations, Fisher exact test *P* = 0.002; CDKN2A/B copy number loss, Fisher exact test *P* = 0.0001; HLA LOH, Fisher exact test *P* = 0.0007). This same cluster of patients, which we refer to herein as “ITH-high”, had comparable levels of tumor purity to the other “ITH-low” cluster, but demonstrated elevated ITH at the level of somatic DNA alterations, RNA, and TCR (combined Fisher exact test *P* = 0.0495). Moreover, by classifying patients into previously described ccRCC evolutionary subtypes (Additional file 2: Fig. S3), we observed that PBRM1-driven tumors were enriched in the ITH-high cluster (on sample level, Fisher exact test *P* = 0.0018), in agreement with TRACERx Renal [15]. We did not find any association between ITH and other gene mutations (Fisher exact test *P* > 0.5). However, this finding must be treated with caution due to our relatively small cohort size as well as low number of regions collected in some patients. These findings were robust to the number of regions collected per tumor, and we found no significant association between ITH and exposure to ICI (Fisher exact test *P* = 0.65, Fig. 3B); however, due to the small size of our untreated cohort, this analysis might be underpowered. Together, our results demonstrate that (1) ITH is not restricted to genomic events, but rather is pervasive in the transcriptome, microenvironment, and immune compartment of ccRCC tumors, and (2) correlates with specific somatic events at the level of individual patients (i.e., PBRM1 and SETD2 mutations, HLA LOH and CDKN2A/B loss).

### ITH-high ccRCC tumors are immunologically distinct

Comparing the TME characteristics of ITH-high and ITH-low patients, we observed that ITH-high tumors (defined as all regions belonging to a patient who is classified as ITH-high) were characterized by high myeloid and low T cell effector (Teff) signatures (Fig. 3C). Similarly, a signature associated with antigen presentation (APM) [4] was downregulated in ITH-high patients, consistent with elevated levels of HLA LOH in the ITH-high subtype. To validate if genomic features uniquely characterizing ITH-high tumors (HLA LOH and CDKN2A/B loss) might be more generally associated with myeloid infiltration in a large, independent cohort, we obtained DNA and RNA sequencing data from the TCGA KIRC study and scored samples by the presence of CDKN2A/B loss, ITH (as measured by the number of clones estimated per sample using PhyloWGS, see “Methods”), and myeloid infiltration. This analysis confirmed that in ccRCC, CDKN2A/B loss was associated with higher levels of ITH (*P* = 3 × 10^−5^) and higher myeloid infiltration (*P* = 7 × 10^−5^) (Fig. 3D). However, the association between genomic ITH and myeloid infiltration did not reach statistical significance in TCGA KIRC cohort suggesting the association between myeloid infiltration and ITH is likely indirect through certain genomic events such as CDKN2A/B loss.

The findings above suggested that ITH-high tumors may be distinct in their immunophenotype, including in the diversity of their T cell repertoire. We therefore investigated the association between ITH and T cell diversity both peripherally and within the tumor. To do so, we compared the overlap between tissue-resident and peripheral T cells. Repertoire overlap analysis (Additional file 2: Fig. S4) illustrated a high degree of shared clonotypes across different tumor regions from the same patient, but a lack of shared clonotypes across patients. ITH-high patients demonstrated a significantly lower peripheral TCR diversity, richness, and clone count compared to ITH-low patients (Fig. 3E), suggesting that elevated heterogeneity in the primary tumor is associated with reduced peripheral immunologic diversity in a manner that is consistent with reports in other diseases [49]. However, the association between ITH and TCR diversity remains correlative and future mechanistic studies are required to establish a causal relation between these two features of tumor and immune-phenotype. Together, the above data argue that elevated molecular heterogeneity in ccRCC tumors is associated with a distinct microenvironmental and immunologic phenotype.

### ICI therapy is associated with loss of putative neoantigens and HLA LOH

The clinical management of ccRCC (for which pre-surgical biopsies are often not indicated or used) makes serial profiling of primary tumors on therapy challenging, rendering our understanding of how ICI may remodel tumor physiology incomplete. To overcome this challenge, we took advantage of 16 patients from our neoadjuvant nivolumab clinical trial who had WES performed on their pre-treatment biopsies. This offered a unique opportunity to interrogate both genomic adaptations (including both somatic mutations and the expression of potentially immunogenic endogenous retroviral elements, HERVs) to ICI therapy and immunologic changes in the T cell repertoire.

**Fig. 4:**
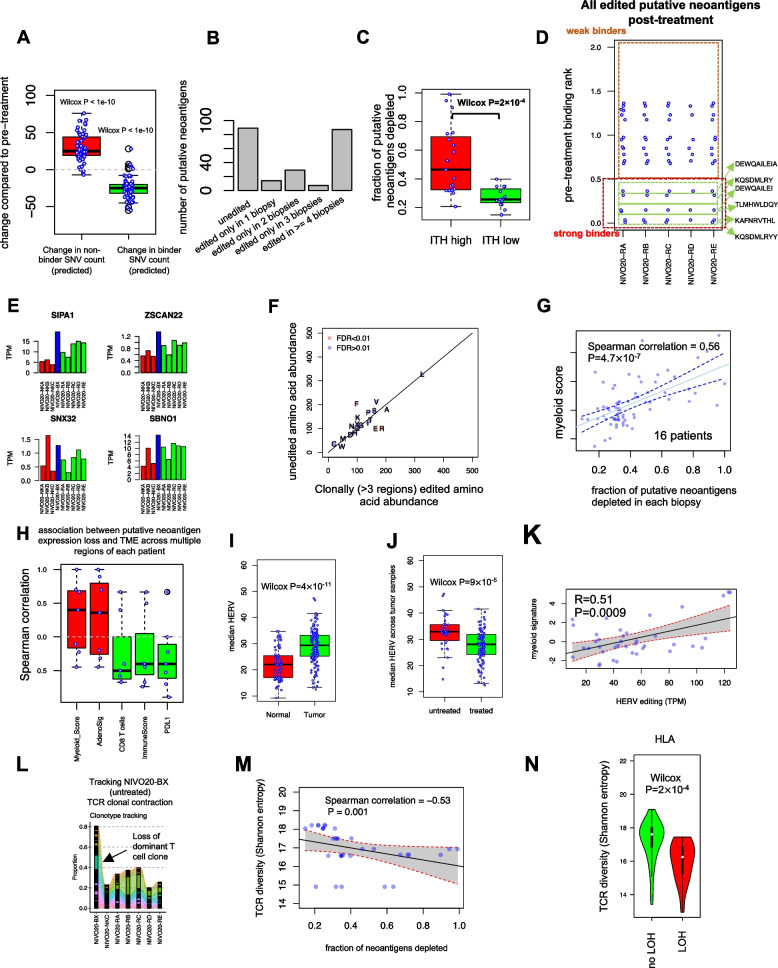
The landscape of heterogeneity of neoantigen depletion. **A** Change in the number of nonsynonymous binder SNVs (predicted in silico) and nonsynonymous non-binder SNVs compared to pre-treatment. Reduction in only putative neoantigens illustrates selective pressure and immunoediting. One sample Wilcox test *P* (compared to zero) is shown. **B** Clonality of neoantigen depletion. Only strong binders are shown. **C** Association between neoantigen depletion and ITH subtypes. **D, E** Immunoediting in an HLA-intact patient NIVO20 through reduced neoantigen expression. NKA/NKB/NKC (shown in RED) are normal adjacent tissues 1, 2, and 4 cm away from the center of the tumor; BX (shown in blue) represents pre-treatment biopsy; RA/RB/RC/RD/RE (shown in green) illustrate 5 tumor regions from the treated tumor sample. **F** Immunoediting with amino acid resolution. Higher phenylalanine (F) depletion compared to glutamic acid (E) and arginine (R) suggests immune selection. **G** Association between putative neoantigen depletion and myeloid activation across all regions of patients where pre-treatment WES data was available (*n* = 16 patients). **H** Association between the fraction of expressed putative neoantigens depleted and immune signatures. In **H**, correlations are calculated across different regions of the same patient, for all patients with > 3 treated, as well as pre-treatment RNA samples were available (*n* = 7 patients). **I, J** HERVs are enriched in tumors compared to normal samples and are associated with treatment. **K** HERV depletion association with myeloid signature. **L** Clonotype tracking of dominant untreated T cell clones in treated regions of patient NIVO20. The color of each ribbon shows different T cell clones, and the width is scaled corresponding to the frequency of that clone. Tissue data consists of 5 tumor regions after treatment (RA/RB/RC/RD/RE), one single normal adjacent (NKC), and one tumor region pre-treatment (BX). Likewise, PBMC data points on treatment are NIVO20-68, -54, -40, -12. **M, N** TCR diversity is negatively associated with neoantigen depletion and HLA LOH

Focusing first on genetic alterations, we anticipated that ICI administration would lead to elimination of some tumor clones and therefore a contraction in total mutation count. However, we observed no consistent trend in the change of either SNV or indel mutational count following ICI therapy (Additional file 2: Fig. S5). Nevertheless, the number of nonsynonymous SNVs that were predicted to bind to MHC complex in silico was consistently reduced across all patients and all biopsies except for NIVO03 (Additional file 2: Fig. S5 and Fig. 4A). An opposite trend was observed in the number of putative non-binders, suggesting a selection in favor of non-neoantigenic mutations by tumor during clonal evolution (Fig. 4A).

In order to characterize the clonality of putative neoantigen depletion across distinct tumor regions, we counted all 8–11 amino-acid-long putative neoantigens seen prior to treatment but deleted in at least one biopsy after treatment. Among 7 patients with at least 4 tumor regions sequenced, we observed an enrichment for putative neoantigen depletion across 4 or more sites (Fig. 4B). To assess the link between ITH and neoantigen depletion, we examined the magnitude of putative neoantigen depletion in each patient by measuring the average number of putative neoantigens deleted per biopsy (i.e., the ratio of the deleted neoantigens in a treated region compared to pre-treatment over the total number of pre-treatment neoantigens). Using this metric, we observed a strong association between neoantigen depletion and ITH subtypes (Fig. 4C). Focusing on patient NIVO20, all 6 identified depleted putative neoantigens were deleted in at least 4 regions, suggesting putative neoantigen depletion is a clonal event (Fig. 4D). Genes expressing these depleted neoantigens demonstrated a 2–threefold reduction in expression related to pre-treatment biopsy (NIVO20-RA/RB/RC/RD/RE vs NIVO20-BX) (Fig. 4E). Together with the data above, these observations suggest that ICI therapy in ccRCC is associated with the clonal loss of mutations with elevated immunogenicity.

Premised on prior reports [50] of the increased immunogenicity of hydrophobic residues, we sought to determine whether a selective pressure exists on certain neoantigens. We compared the number of amino acids preserved versus depleted upon immunotherapy and noticed a strong selection against phenylalanine (F, extremely hydrophobic) in favor of arginine (R, extremely hydrophilic) and glutamic acid (E, extremely hydrophilic) in our cohort (Fig. 4F).

Next to elucidate the link between the TME and neoantigen depletion (Additional file 1: Table S5), we compared different TME gene expression signatures and the fraction of neoantigen depleted. We observed that the fraction of neoantigens depleted was strongly associated with myeloid-high regions (*n* = 16 patients whose pre-ICI treatment WES data was available, Fig. 4G). The association between myeloid activation and neoantigen depletion remained strong when total number of neoantigens depleted was used (instead of fraction) (Additional file 2: Fig. S6B) or when putative neoantigen (transcriptional) expression was taken into account (*n* = 7 patients whose pre-treatment WTS data was available, Fig. 4H) and was not affected by variation in tumor purity (Additional file 2: Fig. S6). Furthermore, the correlation between the degree of neoantigen depletion and myeloid infiltration was also evident when examining different regions of individual patients, where highly depleted regions were associated with the highest myeloid and lowest ImmuneScore (Fig. 4H).

A recent study [51] identified tumor infiltrating lymphocyte-specific HERV epitopes that are translated, can bind to MHC I complex, and induce high-avidity cytotoxic T cells. In [51] as well as other previous reports [52], overexpression of HERVs on tumor cells has been reported and a link to ICI response has been documented [53]. To interrogate other tumor-intrinsic features associated with immune response in our cohort, we utilized our deep RNA sequencing (~ 200 million reads/library) to quantify HERV expression. HERVs were overexpressed in tumors compared to normal tissues in our cohort (Fig. 4I), and median HERV (median of all HERV loci investigated) was correlated to angiogenic expression (Additional file 2: Fig. S8A). Notably, PBRM1 mutations, which lead to further HIF upregulation [54] and angiogenic expression [55, 56], were also positively associated with HERV (Additional file 2: Fig. S8B), consistent with a recent report [57]. In agreement with [53], we then confirmed the association between the median expression of different HERV loci and TIL abundance (Additional file 2: Fig. S8A). Median HERV was anti-correlated with tumor purity; however, the association between HERV expression and TIL abundance remained valid even when HERV expression was corrected for tumor purity (Additional file 2: Fig. S8A). Conversely, we observed a significant reduction in HERV expression an observation akin to reduction in neoantigens (Fig. 4J). Likewise, we observed a strong correlation between HERV editing (i.e., change in the expression of immunogenic HERV loci after treatment, see “Methods”) and myeloid signature further highlighting the association between neoantigen depletion and myeloid enrichment (Fig. 4K). Due to the limitations of HERV quantification using WTS, we could not rule out that a strong correlation between HERV and TIL abundance might be due to expression of HERV on immune cells. However, the expression of HERV on ccRCC tumor cells has been previously shown [58] and their immunogenicity is well-established [51]. Nevertheless, rigorous determination in future studies of cell-specific expression of HERVs will be critical to understanding their putative association with ICI response.

Finally, using TCRseq of tissue-resident and peripheral T cells, we investigated the impact of ICI and neoantigen depletion on T cell diversity. Focusing again on patient NIVO20 where TCR data of multiple regions of pre-treatment and ICI-treated tumor were available, we evaluated the degree of overlap between T cell clonotypes at different regions and time points, i.e., pre-treatment, on-therapy, and post ICI treatment (Fig. 4L). Tracking dominant tissue-resident T cell clonotypes, we noticed a substantial depletion of dominant T cell clones upon ICI therapy (Fig. 4M). This observation was mirrored across our entire cohort, where we observed a strong negative association between peripheral TCR diversity and neoantigen depletion and allele-specific HLA loss across the entire cohort where PBMC TCRseq data was collected (Fig. 4M, N). Together, if validated using future mechanistic experiments, our findings suggest that neoantigen depletion in primary ccRCC tumors is associated with peripheral loss of neoantigen reactive T cells. However, at this point, no causal relationship between neoantigen loss and TCR diversity can be drawn.

### Subclonal evolution underlies immune escape

**Fig. 5:**
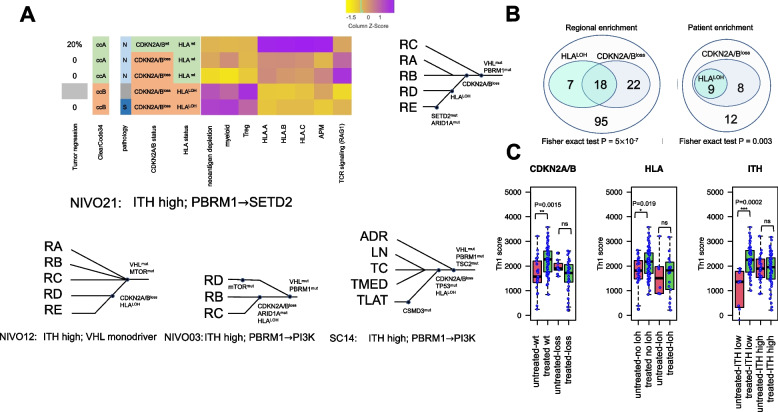
Branch evolution demonstrates immune evasion. **A** Evolutionary tree illustrates tumors can exploit concurrent HLA LOH and CDKN2A/B loss to escape immune surveillance. **B** Co-occurrence of HLA LOH and CDKN2A/B can be seen both across regions and patients. **C** Differential immune response to ICI therapy in patients with CDKN2A/B loss or HLA LOH or belonging to ITH high subtype

In order to understand the immunologic mechanisms driving subclonal evolution after ICI, we investigated in detail patients whose tumors underwent subclonal immunoediting in distinct regions. Strikingly, subclonal reconstruction revealed recurrent subclonal evolution of HLA LOH and CDKN2A/B loss following ICI therapy (Fig. 5A). Notably, we observed HLA LOH and CDKN2A/B loss co-occur in 9 patients (Fisher exact test *P* = 0.003) and most tumor regions (Fisher exact *P* = 5 × 10^−7^) (Fig. 5B). Strikingly, comparing the untreated and treated regions, we only observed a significant immunological response (as measured by Th1 response) in regions without CDKN2A/B loss or HLA LOH (Fig. 5C), suggesting that HLALOH or CDKN2A/B loss are subclonal determinants of response to ICI [38, 44, 48]. This is consistent with recently published data [49] indicating the loss of 9p21—encompassing CDKN2A/B—confers a cold tumor immune microenvironment and resistance to ICI. In that study, Han et al. [49] linked 9p21 loss to a decreased abundance of B, T, CD8 T, NK cells and cytotoxic lymphocytes, and an increased fractions of macrophages, as well as reduced TCR CDR3 repertoire abundance and diversity. We interpret our observations to mean that immunoediting occurs under selective pressure by which certain tumor subclones transform to a less immunogenic phenotype through HLA LOH and CDKN2A/B loss, and that this subclonal selection can produce a highly heterogenous TME.

To further shed light on the how tumor evolution can transform TME, we sought to analyze the spatial distribution of TILs within the TME and their interaction with the stromal compartment using immunohistochemical data. Following A.W. Zhang and colleagues [59], a dedicated genitourinary pathologist classified tumor regions into 3 subtypes according to the co-localization of tumor infiltrating lymphocytes and tumor cells based on lymphocyte morphology: N-TIL (tumors sparsely infiltrated by TILs), S-TIL (tumors dominated by stromal TILs), and ES-TIL (tumors with substantial levels of both epithelial and stromal TILs) (Additional file 2: Fig. S9, Additional file 1: Table S6). We observed that an ES-TIL enriched TME is strongly associated with regions with HLA LOH (ES = 4, N = 7, S = 5 compared to ES = 2, N = 32, S = 21 in HLA-intact regions, Fisher’s exact test *P* = 0.036) or loss of CDKN2A/B (ES = 4, N = 7, S = 9 compared to ES = 2, N = 32, S = 17 in regions without loss of CDKN2A/B, Fisher’s exact test *P* = 0.03) whereas N-TIL pathology is linked with regions with no HLA LOH and no CDKN2A/B loss across the cohort. These findings suggest that despite abundant TILs, post ICI ES-TIL are associated with tumor clones that have evolved genetic mechanisms for evasion of the immune response (HLA LOH and/or CDKN2A/B loss). However, future mechanistic studies are needed to pinpoint the primary genomic event that transforms the ccRCC TME into a cold niche.

### An adverse ccRCC TME is enriched stroma and myeloid signatures

**Fig. 6:**
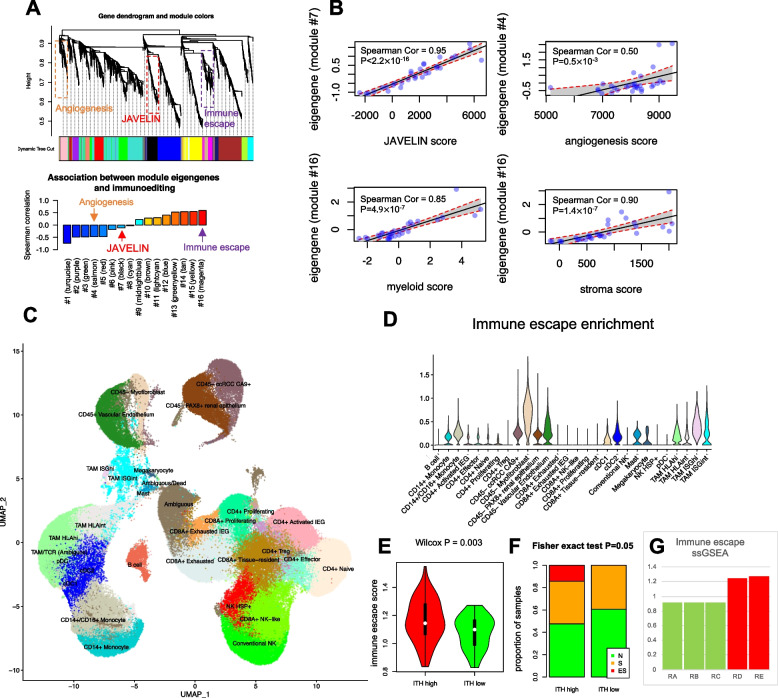
Immunoediting correlates with stroma and myeloid signatures. **A)** WGCNA identifies gene expression modules associated with inflammation (“JAVELIN-like”), angiogenesis, and Immune Escape. Gene dendrogram was first generated and then modules were extracted using dynamic tree cutting (top). Modules were annotated by comparing the correlation between the module eigengenes and previously known gene signatures describing different phenotypes (bottom). **B** Modules 7 (black), 4 (salmon), and 16 (magenta) are associated with previously described signatures, JAVELIN, angiogenesis, and myeloid/stroma. **C** scRNAseq demonstrates the cell type enrichment of Immune Escape signature in ccRCC patients. Different colors represent different cell types inferred from scRNAseq data. UMAP plot illustrates single cells collected from all 6 patients including treated and untreated patients. Computational extracted clusters were annotated as previously described [15]. **D, E** Association between ITH groups, Immune Escape signature, and N/S/ES pathologies. **F** These regions demonstrate an elevated Immune Escape gene signature in NIVO21. RA, RB, RC, RD, and RE denote different regions of a tumor sample

We hypothesized that neoantigen depletion could be associated with a specific transcriptional signature, akin to those identified in clinical trial settings as biomarkers for response to ICI in ccRCC. To identify such a signature, we performed unsupervised Weighted Gene Co-expression Network Analysis (WGCNA) [60] to reconstruct modules from our transcriptomic samples similar to [11] (Fig. 6A). Reassuringly, we identified two gene expression modules #7 and #4 reflecting established microenvironmental features associated with therapeutic response in ccRCC: immune inflammatory response (“JAVELIN-like” signature) and “angiogenesis-like” (Fig. 6A, B). We next assessed the correlation between the expression of each WGCNA gene module and neoantigen depletion. While the JAVELIN-like and angiogenesis-like modules showed no association with neoantigen depletion, module 16 demonstrated the strongest association (Fig. 6A). Correlation analysis with previously known gene expression signatures illustrated that module 16 (which we refer to as an “*Immune Escape*” signature) was strongly associated with myeloid and stroma features of TME. The Immune Escape signature also resembled a recently described pan-cancer TGF-β signature derived in a previous study [45] which was linked to cancer-associated fibroblasts enriched in immune evasion and immunotherapy failure. However, no association between the Immune Escape signature and treatment status was observed (Wilcox *P* = 0.79) (Additional file 2: Fig. S10).

To reveal the primary cellular populations driving the Immune Escape signature in the ccRCC TME, we leveraged scRNAseq from multiple tumor regions, lymph node, normal kidney, and peripheral blood of two ICI-naïve and four ICI-treated patients [16] (*n* = 167,283 single cells). We identified 28 clusters (Fig. 6C) using Louvain clustering [61, 62], and each cluster was annotated based on our previous study [16]. As expected, scRNAseq revealed enrichment of this signature in renal epithelium, tumor stroma, and tumor-associated macrophages (TAMs) and monocytes (Fig. 6C). Hence, scRNAseq, histopathological evaluation, and immunofluorescence (Additional file 2: Fig. S10B) further confirmed the association between Immune Escape and neoantigen depletion (Fig. 6A, Spearman correlation = 0.6), ITH (Fig. 6D, Wilcox *P* = 0.003), myeloid activation (Fig. 6B, Spearman correlation = 0.8, Additional file 2: Fig. S10B) and with stroma, and renal epithelium histopathology (Additional file 2: Fig. S9 and Fig. 6E, F).

### Immune Escape correlates with clinical outcome to ICI therapy

Several previous studies have associated signatures of Immune Escape with poor clinical outcome in ICI-treated patients [63]. Thus, we evaluated whether our Immune Escape signature can correlate with clinical outcome to ICI treatment. We obtained publicly available RNAseq data for several clinical trials including phase 3 JAVELIN Renal 101 trial [11]—a phase III randomized anti-PD-L1 (avelumab) plus tyrosine kinase inhibitor (TKI, axitinib) versus multi-target TKI (sunitinib), IMmotion151 [64]—a phase III trial comparing anti-PDL1 (atezolizumab) plus anti-angeniogenesis agent (bevacizumab) versus TKI (sunitinib) in first-line metastatic renal cell carcinoma, CheckMate 009/010—a phase I/II, aPD-1 (nivolumab) treated, and CheckMate 025—a phase III randomized mTOR inhibitor (everolimus) versus aPD-1 [10]. We stratified patients by the median score (see “Methods”) of the 3 gene signatures obtained in our study (i.e., module 4/JAVELIN_like, 7/angiogenesis-like, and 16/immune escape).

**Fig. 7:**
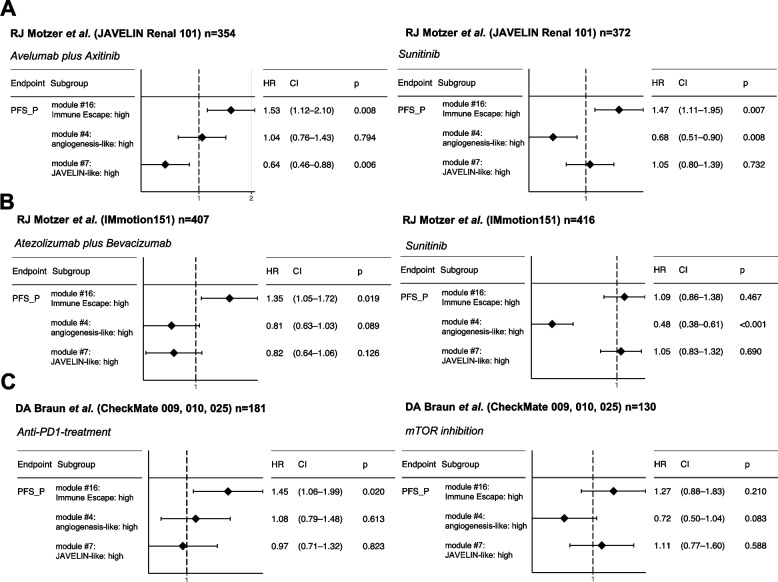
Association between immune escape and clinical outcome to checkpoint blockade. Survival analysis shows the association between gene signatures obtained in this study and clinical outcome of different independent retrospective trials. **A** Immune Escape and JAVELIN-like signatures are associated with PFS in patients treated Avelumab plus Axitinib in JAVELIN Renal 101 cohort. **B** Immune Escape signature, but not the JAVELIN-like signature, correlates with the response to atezolizumab plus bevacizumab in IMmotion151 but not JAVELIN signature. **C** Immune Escape signature, but not the JAVELIN-like signature, correlates with the efficacy of anti-PD1-treament in CheckMate 009, 010, 025

The Immune Escape signature was strongly associated with the response to all three ICI regimens (avelumab plus axitinib HR = 1.53 *P* = 0.008, atezolizumab plus bevacizumab HR = 1.35 *P* = 0.019, and nivolumab HR = 1.45 *P* = 0.02, Fig. 7 and Additional file 2: Fig. S11). In contrast, the JAVELIN-like inflammatory signature was strongly associated with clinical outcome to avelumab plus axitinib (HR = 0.64 *P* = 0.006), but no association with clinical benefit was found between atezolizumab plus bevacizumab (HR = 0.82 *P* = 0.126) or nivolumab treatment (HR = 0.97 *P* = 0.823) (Fig. 7). Similarly, the angiogenesis-like signature was strongly correlated with the response to sunitinib in both IMmotion151 (HR = 0.48 *P* < 0.001) and JAVELIN Renal 101 (HR = 0.68 *P* = 0.008) as expected, but not with ICI-associated regimens. Associations between the Immune Escape signature and therapeutic response remained valid even when thresholds other than median were used to define immune escape high and low (Additional file 2: Fig. S12). Even though the Immune Escape signature was also associated with response to sunitinib in JAVELIN Renal 101, no association between sunitinib response or mTOR inhibition was observed in IMmotion151 and CheckMate 025. Overall, this analysis suggests that a transcriptional signature associated the tendency to lose putative neoantigens after ICI is associated with response to combination ICI therapy and nominates a new potential biomarker for this therapeutic regimen.

## Discussion

Here we used spatiotemporal, multimodal profiling to investigate the link tumor genomics, microenvironmental heterogeneity, peripheral immune response, and eventual immune escape in advanced and metastatic ccRCC. The fundamental discovery of our analysis is that ITH manifests well beyond the tumor genome and produces highly heterogeneous immune microenvironments in the tumor.

Emerging data on biomarkers of response to ICI in ccRCC has identified two potentially paradoxical observations: first, that TIL abundance alone is an insufficient predictor of ICI response [9], and second, that the presence of myeloid cells correlate with resistance to both ICI and anti-VEGF treatments. Strikingly, we observed that high myeloid score tumors were associated with neoantigen depletion which could, in principle, render ICI treatment ineffective. In agreement with this, we derived a transcriptomic signature associated with neoantigen depletion and Immune Escape, which was expressed in renal epithelium, tumor stroma as well as tumor-associated macrophages (TAMs) and monocytes. This Immune Escape signature was associated with response to several ICI regiments in published clinical trials. In total, these findings suggest that myeloid cells are associated with tumor clones that have evolved mechanisms to escape anti-tumor immune responses. Critically, such a hypothetic model requires detailed work and mechanistic validation in immunocompetent systems, which we are actively developing.

**Fig. 8:**
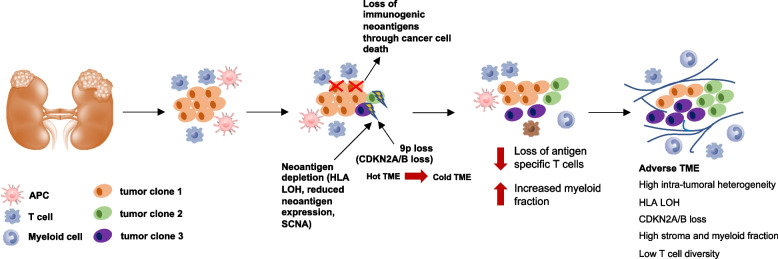
A hypothetical model for spatiotemporal evolution of ccRCC links ITH to immune escape and adverse TME. Cancer cell death, potentially by cytotoxic killing, early in tumor evolution selects for tumor clones with HLA LOH and/or CDKN2A/B loss. This promotes the evolution of a TME depleted of antigen-specific T cells and enriched for myeloid cells

Why do regions with neoantigen depletion demonstrate elevation of myeloid cells but not cytotoxic T cells that would presumably eliminate tumor clones? Cancer immunoediting proceeds through three phases: elimination, equilibrium, and escape [65]. Throughout these phases, tumor immunogenicity evolves, and thereby, despite possible initial response to therapy, acquires immunosuppressive mechanisms that may enable disease progression. Our data suggests that myeloid-high, stroma-enriched (Fig. 6D), neoantigen-depleted tumor regions historically experienced a cytotoxic T cell response, which prompted the selection of tumor clones losing neoantigens and/or HLA/CDKN2A/B. Such a loss of target antigens through HLA LOH or neoantigen depletion would result in loss of antigen-TCR stimulation, leading to death of the corresponding neoantigen reactive T cells (Fig. 8). The selection of hydrophobic residues during the course of neoantigen depletion is another intriguing finding of our study. As demonstrated by others [49], hydrophobicity can result in an increased immunogenicity of the neoantigens. In agreement with this notion, we showed that the hydrophobic epitopes are the most frequently depleted neoantigens likely due to the higher immunogenicity. Likewise, HERVs were pruned by immune selection which might imply that HERVs are associated with immune recognition of HERV expressing tumor cells and thus, ICI-treated regions of the tumor may reduce HERV expression. Importantly, as with other findings in this analysis, the association between neoantigen loss and myeloid activation observed in our data remains purely correlative, and future studies will be necessary to mechanistically establish how immune evasion spatiotemporally evolves in ccRCC following ICI therapy.

Our multi-regional data also has significant implications for biomarker development. We demonstrated that TME markers of response such as JAVELIN and myeloid scores can be heterogenous within tumor regions (Fig. 2, Additional file 1: Additional file 2: Fig. S13). This underscores the importance of accounting for ITH when these signatures are used for patient selection for a specific therapy and longitudinal monitoring of therapies. Given recent data that ICI may have a role in adjuvant therapy following nephrectomy for high-risk disease, our data would suggest that several regions of the primary tumor should be sampled specially in the presence of ITH-associated genomic alterations (e.g., HLA LOH and CDKN2A/B loss). An intriguing finding was a trend towards lower ITH in ICI-treated tumors, even though this observation did not reach statistical significance. If validated in other studies, this in part can be attributed to outgrowth of few nonimmunogenic tumor subclones that managed to escape immune surveillance upon ICI treatment.

An important limitation of this study is that TME heterogeneity of metastatic disease was not assessed and may be less of an issue in biomarker development. Our study has several other potential limitations including its small sample size. To overcome this shortcoming, we validated several of our major findings in several independent cohorts. Another potential limitation of our study is the unavailability pre-treatment multi-regional sequencing data. However, inclusion of multi-regional data from 6 untreated patients allowed us to account for ITH in untreated tumors. Moreover, our neoadjuvant cohort was treated with single agent nivolumab over a short course which may not reflect the TME, and genomic changes induced by more potent combination strategies. Finally, we portrayed the characteristics of an adverse TME which may contribute to ICI resistance. Our study clearly demonstrates the interplay between genomic events and TME transformation from a cytotoxic to a cold immunophenotype. However, these findings remain purely an association of several contributing factors to ICI resistant and the exact causative hierarchy of events requires further investigation.

## Conclusions

Our findings clearly suggest that the ccRCC genome and microenvironment co-evolve, and that loss of putative neoantigens (including SNVs, indels, and HERVs) is associated with a qualitatively myeloid-high environment and the loss of HLA and CDKN2A/B. These distinct genomic alterations are also associated with more peripheral changes, i.e., reduced T cell clonal diversity in the peripheral circulation. In conclusion, we find distinct genomic event enriched in immune escape tumor microenvironment in ccRCC both across and within tumors. Our findings have implications for future biomarker development for ICI response across ccRCC and other solid tumors.

## Supplementary Information

**Additional file1 Supplementary Tables**: Cohort characteristics, gene signature scores, ITH classification and histopathology review results

**Additional file2 Supplementary Figures**: Provides additional data analysis supporting claims and conclusions drawn throughout the paper

## Data Availability

No new code was generated. All data generated in this study are provided in Additional file 1 and Additional file 2. Raw sequencing reads are available from the database of Genotypes and Phenotypes (dbGaP) phs003079.v1.p1 (https://www.ncbi.nlm.nih.gov/projects/gap/cgi-bin/study.cgi?study_id=phs003079.v1.p1) [66] for WES, WTS, and TCRseq.

## Abbreviations

TCGA: The Cancer Genome Atlas
WES: Whole-exome sequencing
WTS: Whole-transcriptome sequencing
SCNA: Somatic copy number alterations
LOH: Loss of heterozygosity
ITH: Intratumoral heterogeneity
TCR: T cell receptor
ccRCC: Clear cell renal cell carcinoma
TME: Tumor microenvironment
ICI: Immune checkpoint inhibitor
TKI: Tyrosine kinase inhibitor
HERV: Human endogenous retrovirus
PBMC: Peripheral blood mononuclear cells
TIL: Tumor infiltrating lymphocytes
WGCNA: Weighted gene co-expression network analysis
ssGSEA: Single-sample gene set enrichment analysis
NKA: Normal kidney 1 cm away from tumor
NKB: Normal kidney 2 cm away from tumor
NKC: Normal kidney 4 cm away from tumor
RA: Region A of tumor (tumor center)
RB: Region B of tumor (tumor superolateral)
RC: Region C of tumor (tumor inferolateral)
RD: Region D of tumor (tumor superomedial)
BX: Before treatment tumor region
TC: Tumor center
TN: Tumor region nearest to kidney
TF: Tumor region furthest from kidney
TEM: Tumor medial
TLAT: Tumor lateral
LN: Lymph node
ADR: Adrenal gland with tumor
TUP: Tumor region upper
TLOW: Tumor region lower
TMED: Tumor region medial
