# Supplementary material for: Spatiotemporal evolution of the clear cell renal cell carcinoma microenvironment links intra-tumoral heterogeneity to immune escape
Source: Genome Med. 2022 Dec 19;14:143. doi: 10.1186/s13073-022-01146-3 (PMC9762114; doi:10.1186/s13073-022-01146-3)
Supplement: Supplementary file 2 — Additional file2 Supplementary Figures: Provides additional data analysis supporting claims and conclusions drawn throughout the paper [file 13073_2022_1146_MOESM2_ESM.pdf]

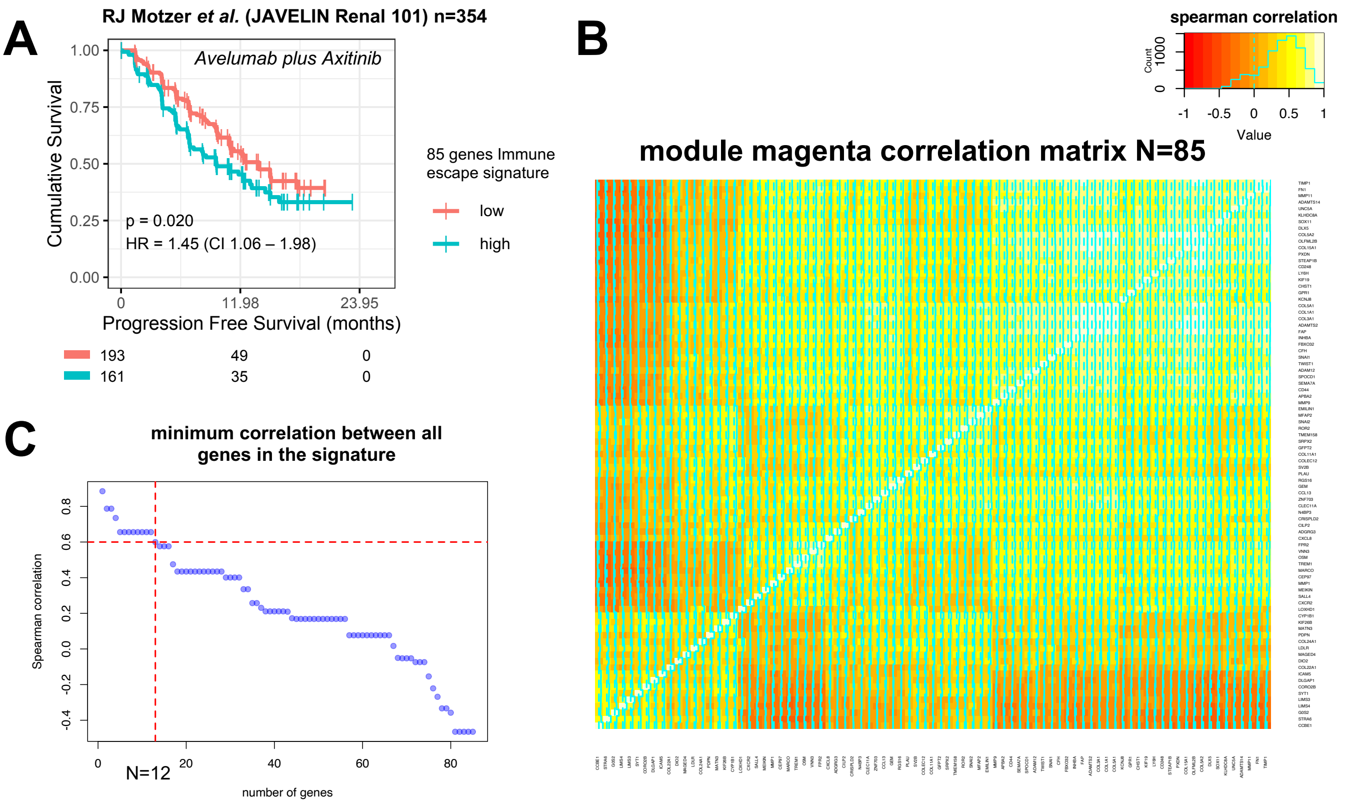

Fig. S1. Refinement of immune escape gene signature. A) 85 genes immune escape gene signature is strongly associated with response to Avelumab plus Axitinib in JAVELIN Renal 101 trial. **B)** Pairwise spearman correlation between 85 genes in module 16 (immune escape). **C)** Refinement of 85 genes into 12 genes with the highest pairwise spearman correlation.

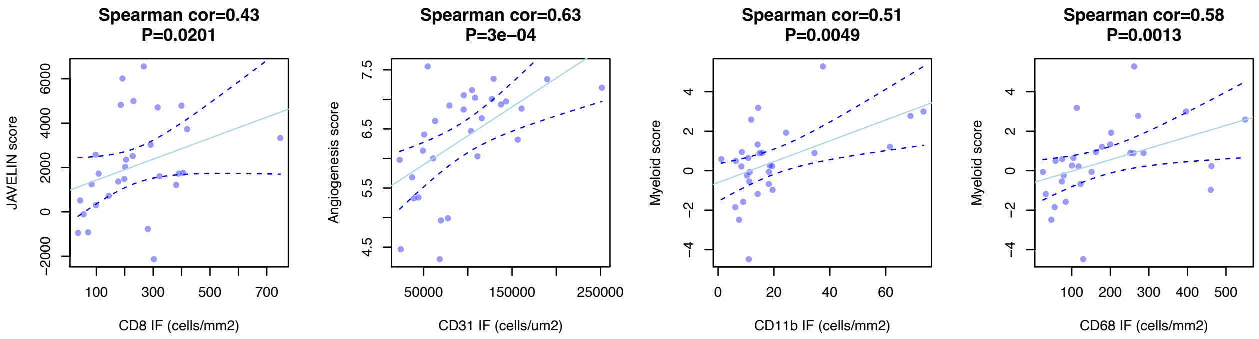

Fig. S2. Validation of TME associated gene signatures using IF. Myeloid signature correlates with CD11b/CD68 markers. CD31 endothelial and CD8 T cell markers are correlated with Angiogenesis and JAVELIN signatures.

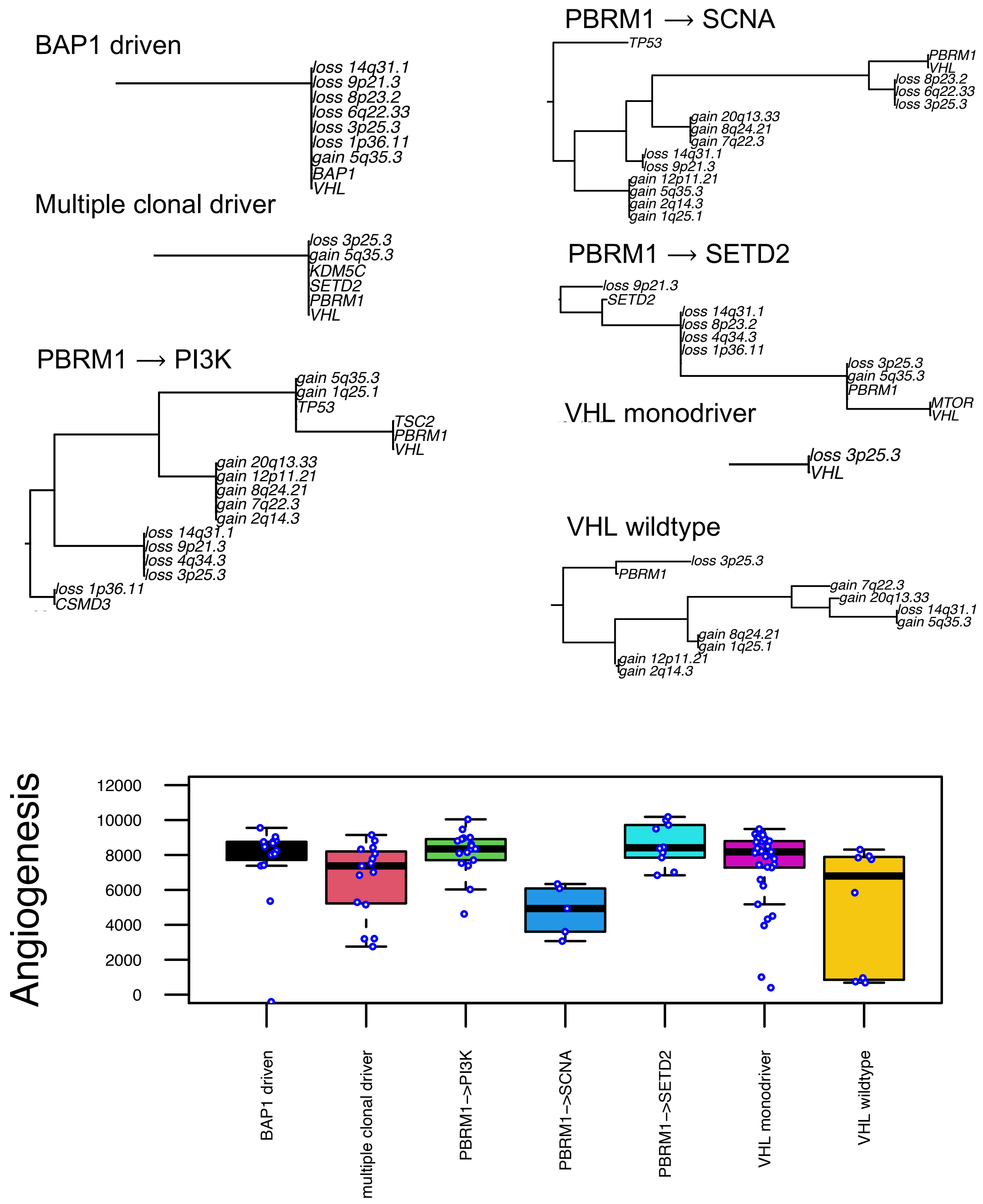

Fig. S3. ccRCC evolutionary subtypes and their association with angiogenic TME score.

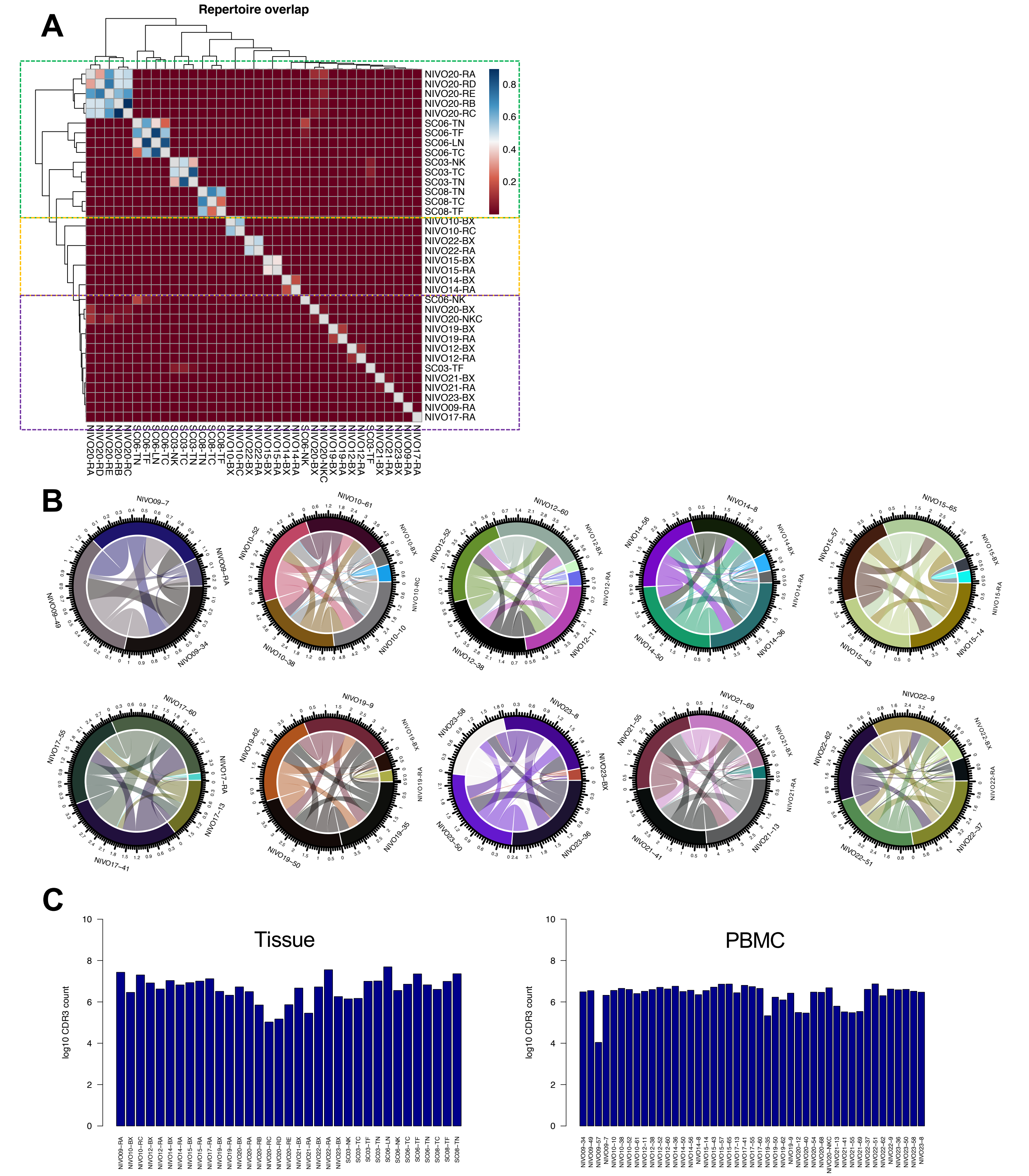

Fig. S4. Clonotype overlap analysis. A) Hierarchical clustering of TCR clonotypes across different regions of patients where tissue TCRseq data was available. **B)** Circos plot illustrates the fraction of shared T cell clonotypes between tissue and different time points on therapy. Ribbons connecting different regions of the tumor are scaled based on clonotype overlap. Labels show patient ID followed by time to nephrectomy (e.g., NIVO09-7: patient NIVO09 at 7 days to nephrectomy). **C)** Number of CDR3 clone counts shown for all samples where TCR seq was performed.

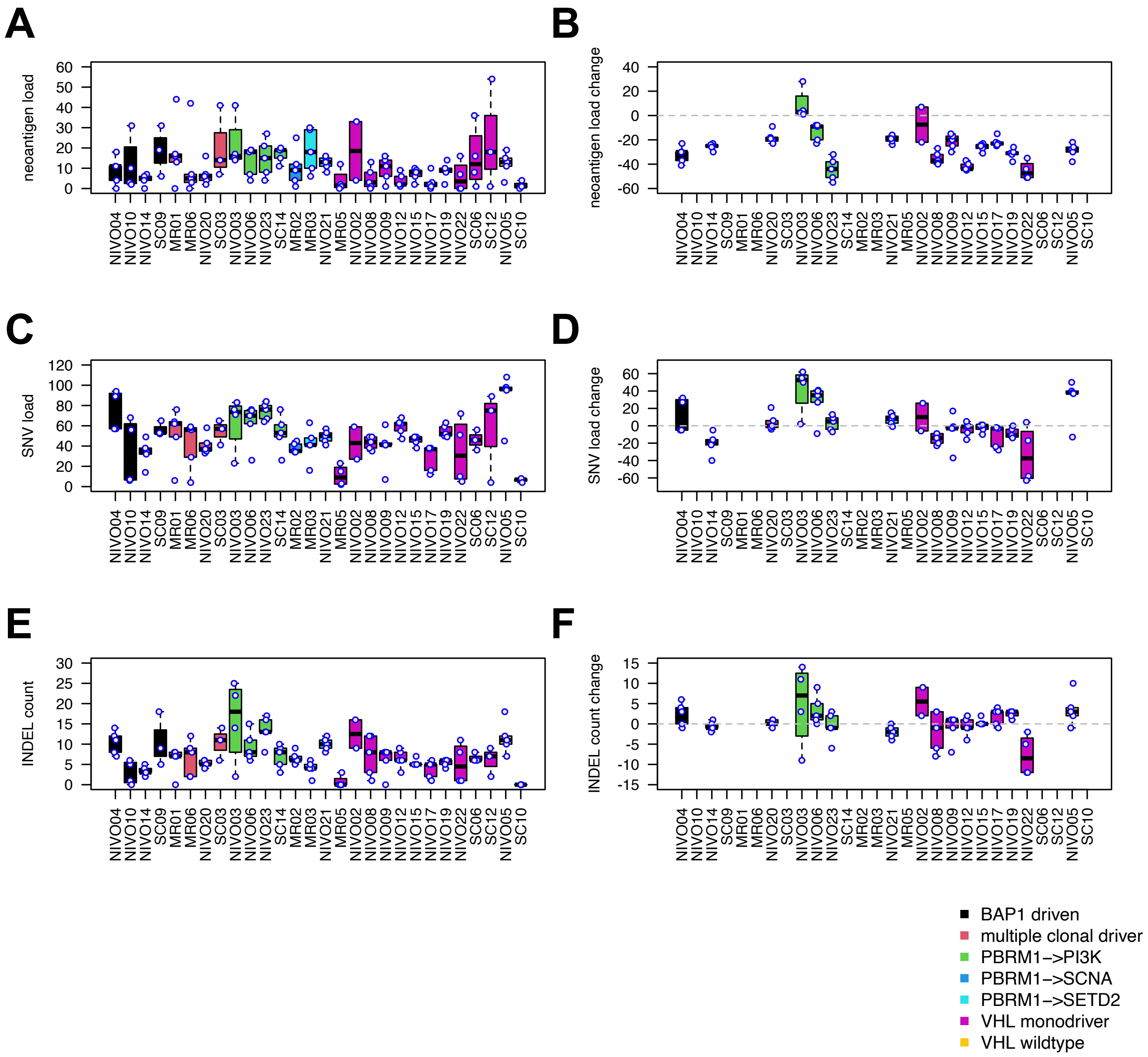

Fig. S5. Boxplots show total and change compared to pre-treatment (when sample was available) for mutational count, and neoantigen count across different regions of all patients. Count change is shown only for 16 patients whose pre-treatment WES data was available.

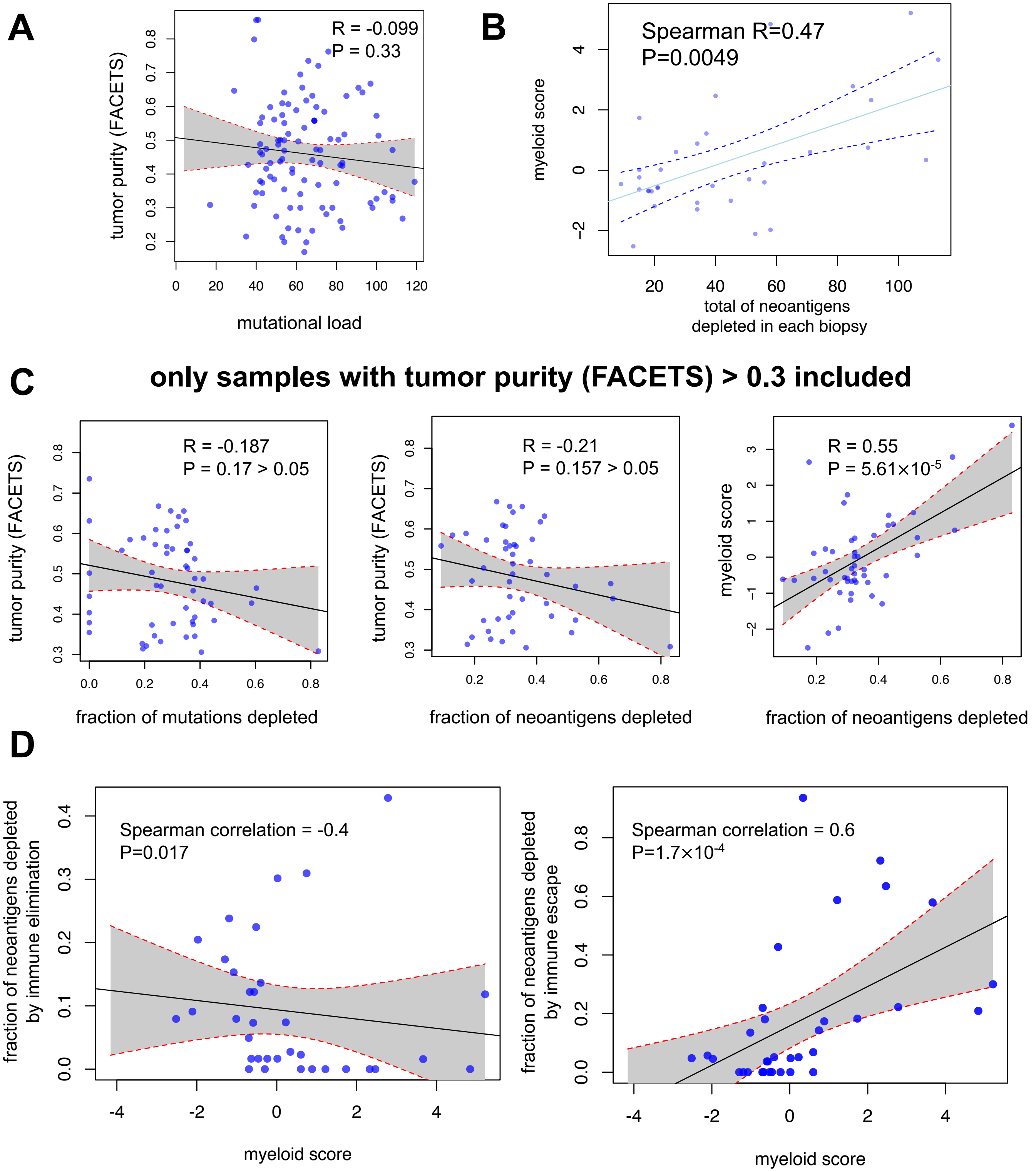

Fig. S6. Association between neoantigen loss and myeloid signature. A) No association between TMB and tumor purity (FACETS) was observed **B)** Association between total number of neoantigens depleted and myeloid signature. **C)** The fraction of mutations or neoantigens depleted is not correlated with tumor purity for samples with tumor purity larger than 0.3; however, the association between myeloid signature and neoantigen depletion remains strong even after excluding samples with low purity. **D)** Association between immune elimination and escape with myeloid signature.

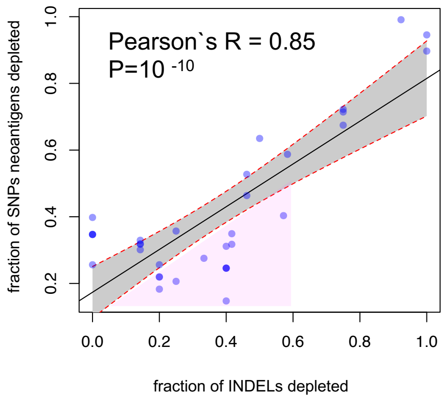

Fig. S7. Comparison between SNPs neoantigen depletion and INDEL depletion.

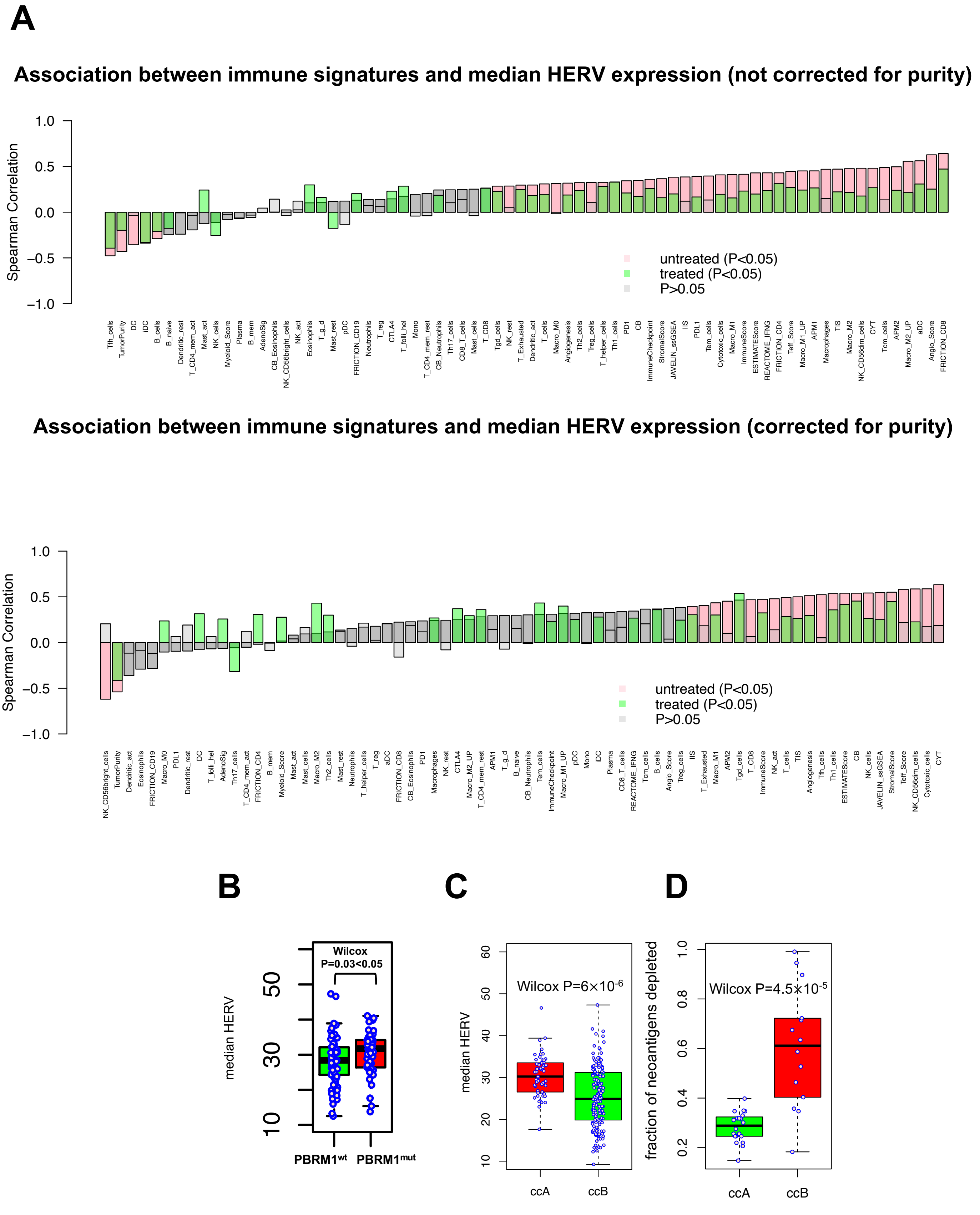

Fig. S8. Treatment impact of HERV association with TME. A) Association between HERV expression and immune signatures. **B)** PBRM1 mutations are associated with elevated HERV expression**. C)** Association between ClearCode34 classes and HERV expression. **D)** Association between ClearCode34 classes and neoantigen depletion.

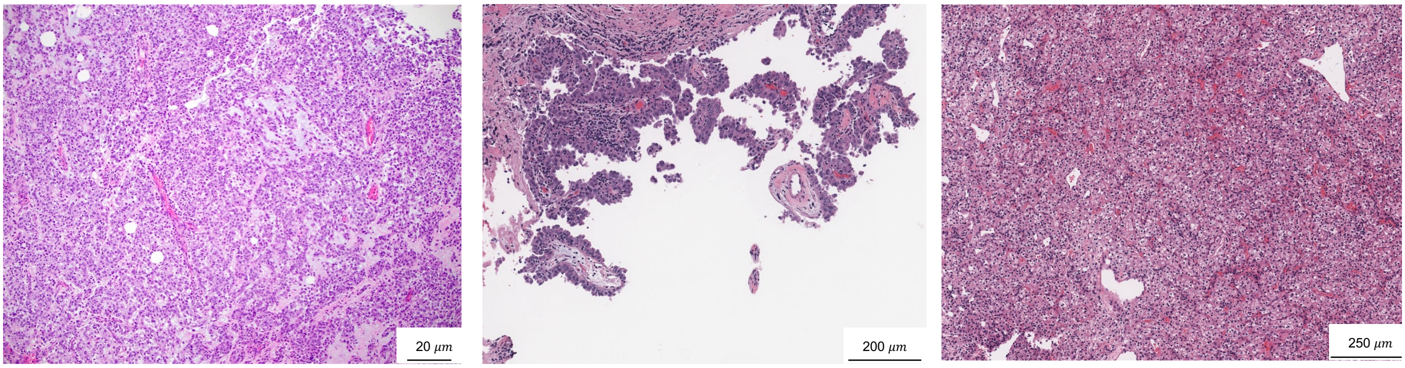

N-TIL (tumors sparsely infiltrated by TILs)

S-TIL (tumors dominated by stromal TILs)

ES-TIL (tumors with substantial levels of both epithelial and stromal TILs)

Fig. S9. Representative histopathology images of different histopathological TIL subtypes. Regions of tumor associated with immune escape depict a distinct pathology where colocalization of TILs and stroma can be observed. N-TIL (tumors sparsely infiltrated by TILs), S-TIL (tumors dominated by stromal TILs), and ES-TIL (tumors with substantial levels of both epithelial and stromal TILs).

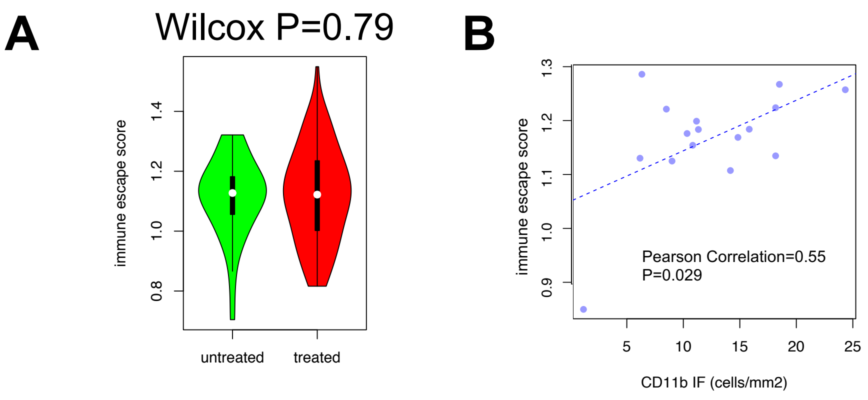

Fig. S10. Supporting data on immune escape signature. A) Association between immune escape signature and treatment. **B)** Association between immune escape signature and myeloid enrichment shown by IF. Only samples from multi-regional cohort included to avoid batch effect.

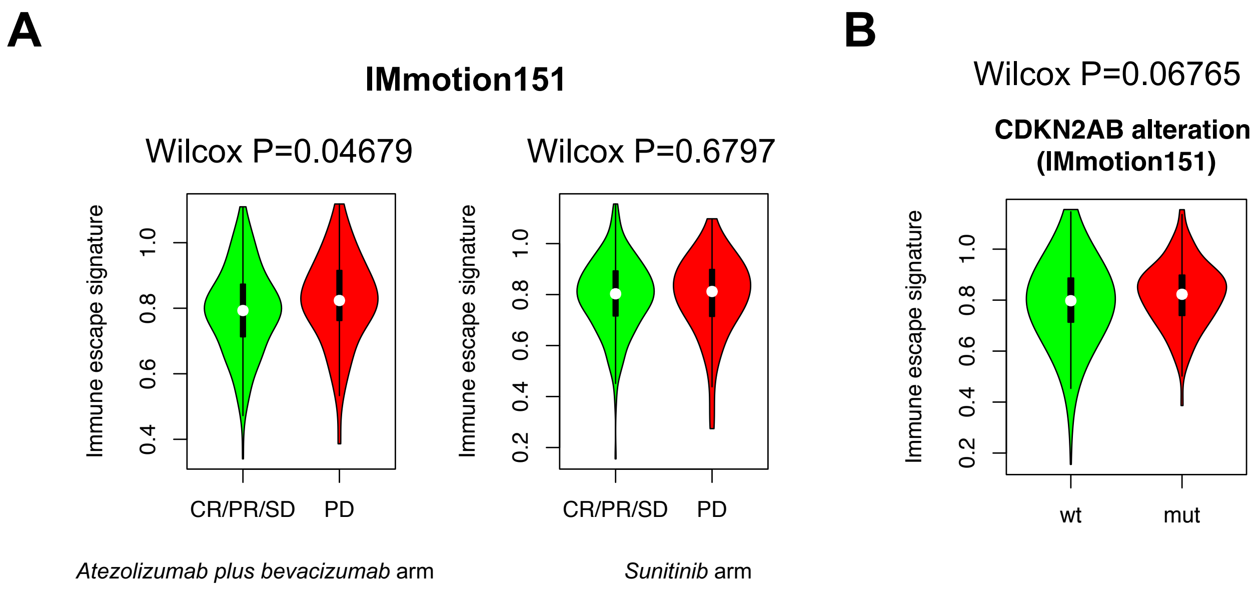

Fig. S11. Validation of escape signature in independent cohorts (IMmotion151). A) Escape signature is associated with improved survival in patients treated with ICI but not sunitinib**. B)** Escape signature is associated with CDKN2A/B alteration in Immotion151.

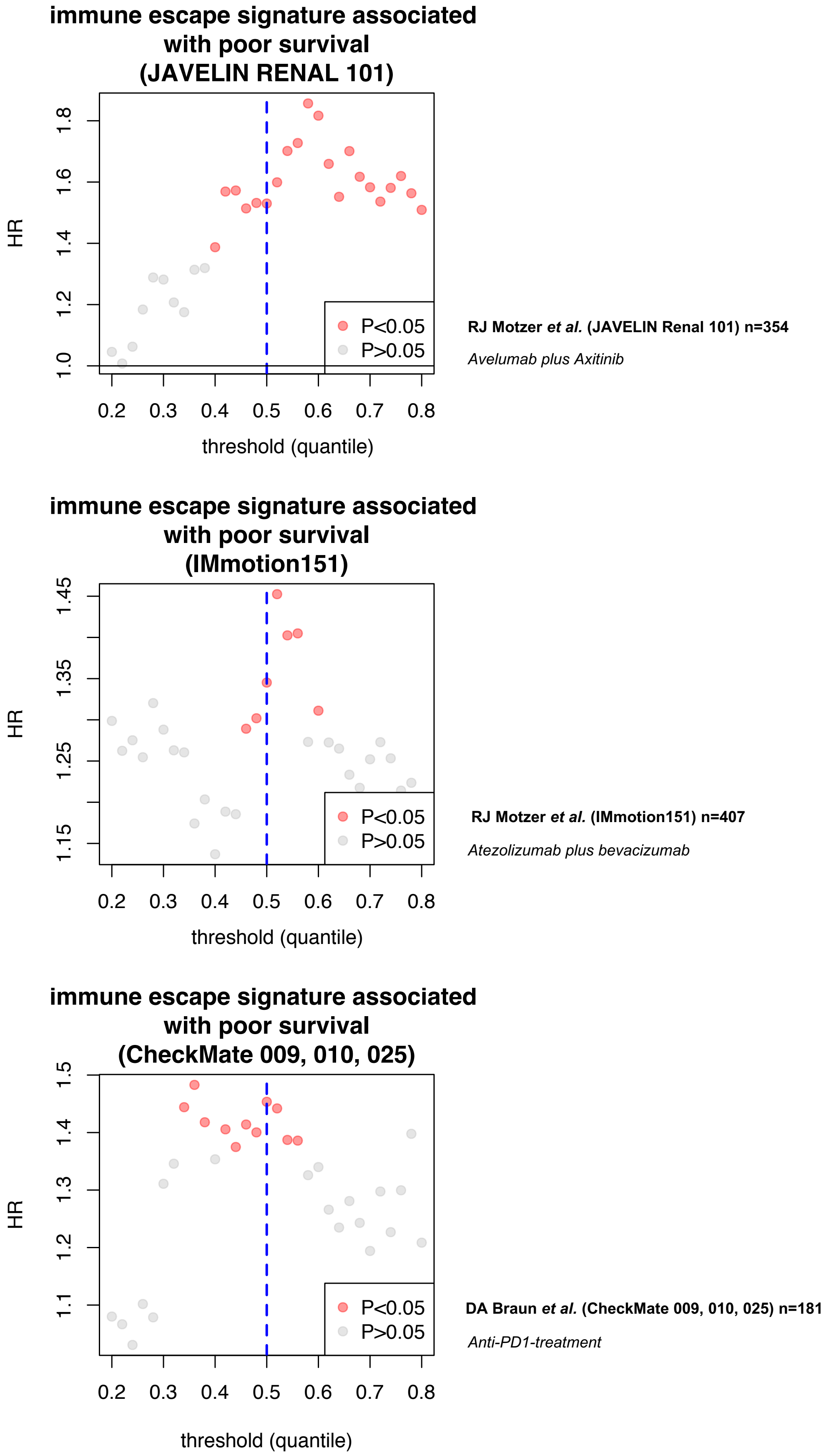

Fig. S12. Relationship between escape gene signature and treatment outcome in different clinical trials. HRs are calculated for each threshold for ICI or ICI in combination with TKI arms in JAVELIN Renal 101, IMmotion151, and CheckMate 009, 010, 025.

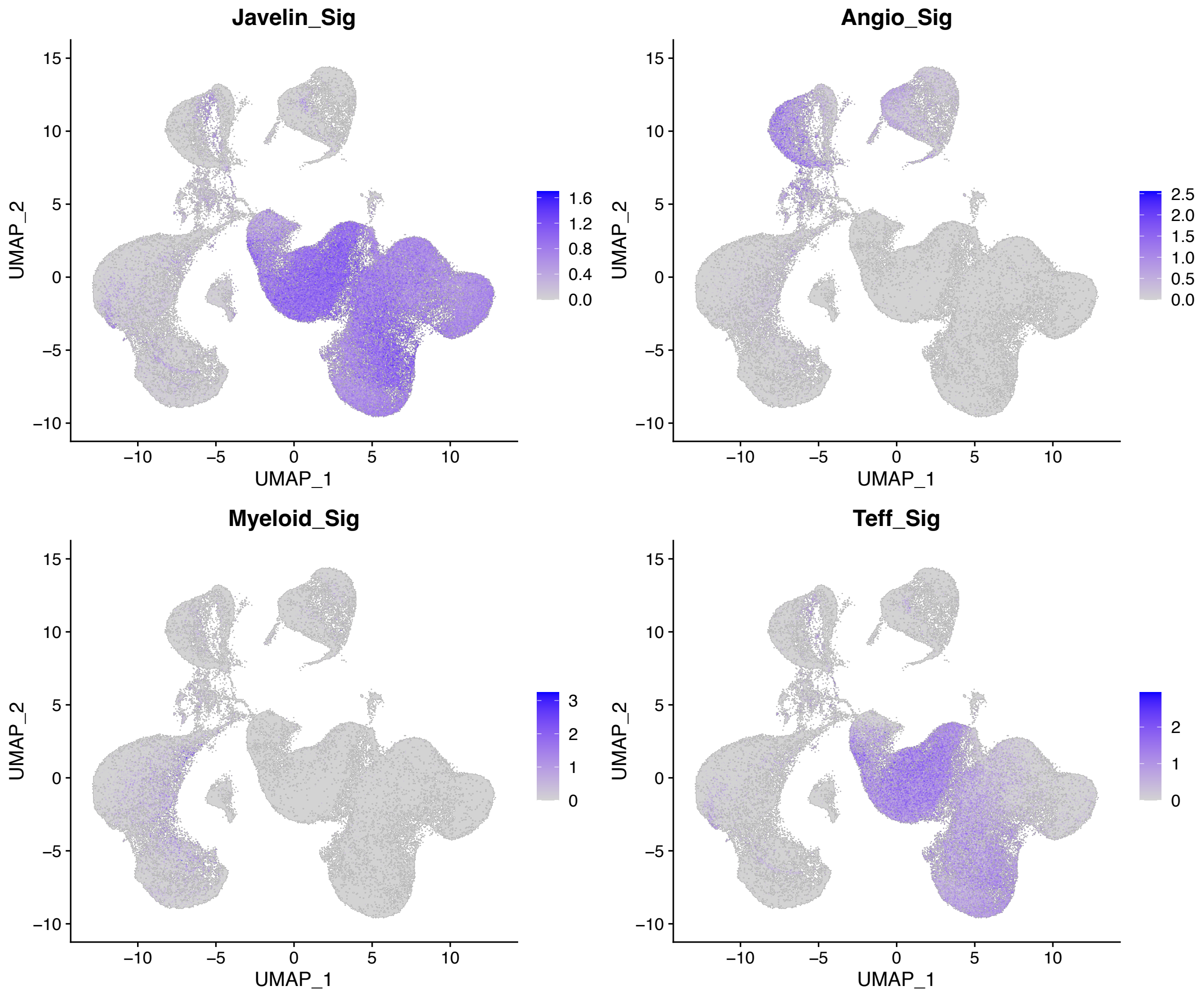

Fig. S13. ITH of 4 commonly used ccRCC gene expression signatures demonstrated using scRNAseq data.
